# A Generalized Mechanistic Codon Model

**DOI:** 10.1093/molbev/msu196

**Published:** 2014-06-23

**Authors:** Maryam Zaheri, Linda Dib, Nicolas Salamin

**Affiliations:** ^1^Department of Ecology and Evolution, Biophore, University of Lausanne, 1015 Lausanne, Switzerland; ^2^Swiss Institute of Bioinformatics, Genopode, Quartier Sorge, 1015 Lausanne, Switzerland

**Keywords:** codon models, phylogenetics, multiple substitutions, positive selection, Markov model, Kronecker product

## Abstract

Models of codon evolution have attracted particular interest because of their unique capabilities to detect selection forces and their high fit when applied to sequence evolution. We described here a novel approach for modeling codon evolution, which is based on Kronecker product of matrices. The 61 × 61 codon substitution rate matrix is created using Kronecker product of three 4 × 4 nucleotide substitution matrices, the equilibrium frequency of codons, and the selection rate parameter. The entities of the nucleotide substitution matrices and selection rate are considered as parameters of the model, which are optimized by maximum likelihood. Our fully mechanistic model allows the instantaneous substitution matrix between codons to be fully estimated with only 19 parameters instead of 3,721, by using the biological interdependence existing between positions within codons. We illustrate the properties of our models using computer simulations and assessed its relevance by comparing the AICc measures of our model and other models of codon evolution on simulations and a large range of empirical data sets. We show that our model fits most biological data better compared with the current codon models. Furthermore, the parameters in our model can be interpreted in a similar way as the exchangeability rates found in empirical codon models.

## Introduction

The recent advances in high-throughput sequencing techniques are providing researchers with a wealth of genome scale data, in particular for nonmodel organisms, that is creating a surge toward comparative genomics (Nielsen et al. 2005; Anisimova and Liberles 2007; Studer et al. 2008; Barrett et al. 2009; Jing et al. 2010; Castoe et al. 2011; Dufresne and Jeffery 2011; Oh et al. 2012; Servin et al. 2012; Huang et al. 2013). Phylogenetic trees are a key element in this comparative framework, and their role has been strengthened by recent and new theoretical developments (Larget and Simon 1999; Ronquist and Huelsenbeck 2003; Felsenstein 2004; Yang 2006; Blanquart and Lartillot 2008; Lartillot et al. 2009; Rodrigue et al. 2010; Dib et al. 2014) that enable a better understanding of evolutionary processes occurring among species and genes (Aleshin et al. 2009; Cranston et al. 2009). However, the full potential of these new data sets will only be achieved by the developments of further statistical and mathematical approaches.

The statistical models that are currently in use to study the evolution of molecular data are designed to approximate, in a simplified form, the complex aspects of evolution (Felsenstein 2004). This simplification allows us to understand more easily the key processes at play and identify the major driving forces behind them. Three types of models can be applied to current molecular sequences depending on the underlying data they are meant to analyze. The dimensionality of the parameter space for these models increases from a 4 × 4 substitution matrix for models of nucleotide evolution to 20 × 20 for amino acids and, finally, to 61 × 61 codon models (stop codons being usually excluded). Depending on the type of data and application at hand, all or some of these models are applicable, and it is crucial to select a model that is appropriate and minimizes the potential discrepancies with the true, yet unknown, evolutionary process.

The type of substitutions occurring at the nucleotide level is easily modeled with Markov models because of the relatively simple properties differentiating the nucleotides. Furthermore, they exhibit only four states, which render these models more tractable than amino acid or codon models from a computational point of view (Yang 2006). Their attractiveness due to lower computational complexity is reinforced by their wider applicability to any type of DNA sequences. For protein-coding DNA regions, however, treating the 3-nt positions within codons as independent evolutionary units is an approximation that does not fully account for the inherent biological reality and can potentially mislead phylogenetic reconstructions (Shapiro et al. 2006; Bofkin and Goldman 2007; Seo and Kishino 2008; Christin et al. 2012).

In contrast to nucleotide models, codons or amino acid models can be applied exclusively to protein-coding sequences. The latter models are widely used to reconstruct evolutionary relationships among distantly related species because they entail a lower risk of saturation (Anisimova and Kosiol 2009). However, amino acid models are not easily amenable to mechanistic statistical modeling because of the physicochemical complexity of the relationship between amino acids. Such models are thus typically estimated from a priori defined empirical data sets (Dayhoff et al. 1978; Jones et al. 1992; Whelan and Goldman 2001). The substitution rates between amino acids are thus not estimated directly from the specific data analyzed, which could lead to inaccuracies during the tree reconstruction (Anisimova and Kosiol 2009). Furthermore, statistical comparisons between amino acid and codon models indicate that synonymous substitutions are very informative (Salamin et al. 2005; Seo and Kishino 2008; Christin et al. 2012). Codon models may then be more appropriate than amino acid models for phylogenetic inference even for highly divergent species.

Unlike nucleotide models that have at most six substitution rates, a fully generalized symmetric codon model would require to estimate 1,830 substitution rates (note that one substitution rate is usually fixed). It is statistically and computationally difficult to fit such a large number of parameters, and several approximations have been proposed to reduce this complexity. The implementations of current codon models have thus followed three main routes.

First, mechanistic models reduce the biological complexity by restricting the parameter space to a small set of evolutionary important parameters (Goldman and Yang 1994; Nielsen and Yang 1998; Pond and Muse 2005; Seoighe et al. 2007; Wong et al. 2006). The parameters typically include selection pressure and differential rates of transitions versus transversions (Yang 2006), although different rates of change between nucleotides in the three codon positions have been recently proposed (Zhou et al. 2010). Different implementations of these models exist (Goldman and Yang 1994; Muse and Gaut 1994). They have further been extended to allow variation of selection pressure among codons and among lineages (Yang and Bielawski 2000; Zhang et al. 2005; Murrell et al. 2012), which allows to explore a wide range of hypothesis testing. The advantage of these mechanistic models is to have a low number of parameters and be computationally tractable, although they still require far more computational resources than nucleotide models (Schabauer et al. 2012).

However, an important assumption of most codon models is that only a single nucleotide substitution per codon is allowed within a small interval of time, and double and triple substitutions within a codon are consequently ignored (Yang 2006). This simplification might be unrealistic as multiple instantaneous substitutions have been observed in real DNA sequences. For instance, the best estimates of protein evolution have nonzero instantaneous rates of change between amino acids whose codons differ by more than one nucleotide (Dayhoff et al. 1978; Whelan and Goldman 2001). This could be explained by highly frequent genomic events, such as codon bias or gene conversions, which can make these double or triple substitutions very likely (Aguileta et al. 2004; Whelan and Goldman 2004; Drake 2007; Hershberg and Petrov 2008). The only fully parametric model that considers multiple instantaneous substitutions was developed by Whelan and Goldman (2004). It calculates substitution rate matrices for single-, double- and triple-nucleotide mutation separately using the equilibrium frequency of mutated nucleotides and a transition to transversion rate. The three matrices are then combined to calculate the general rate matrix of codons. Evaluation of this model on a large amount of coding sequences showed that this assumption improved the likelihood performance compared with the existing mechanistic models (Whelan and Goldman 2004). Although the rates of double and triple substitutions have been estimated to be two to three orders of magnitude lower than single substitutions (Chuzhanova et al. 2003; Smith et al. 2003; Whelan and Goldman 2004), these results further highlight the fact that they cannot be neglected (Whelan and Goldman 2004; Doron-Faigenboim and Pupko 2007).

The second approach to introduce more biological realism into codon models is to use empirical information derived from existing databases to estimate the rate of substitutions between codons (Schneider et al. 2005; Zoller and Schneider 2012). It was initially used to align DNA sequences and for homology searching but was extrapolated into a full model by deriving a substitution rate matrix from the probability matrix (Kosiol and Goldman 2005). The advantage of such a model is that it encapsulates the full biological variability present in the sequences used to build the matrix. However, as in the case of amino acid models, the transition probabilities estimated on some set of sequences do not necessarily represent the changes affecting a particular set of sequences. It has further been shown that the estimation of the substitution rates for deeply diverged species resulted in empirical codon substitutions that lacked accuracy due to limitations in the distance estimation (Schneider et al. 2005).

Finally, the third approach is represented by empirical–mechanistic models, which complement the simplification of mechanistic models with a series of important parameters, such as exchangeability rates, that are estimated empirically from large databases. An implementation of such empirical–mechanistic models is the ECM model (Kosiol et al. 2007), which combines the parameters assumed in the mechanistic model M0 (Goldman and Yang 1994) with physicochemical rates of amino acid substitutions estimated from the Pandit database (Whelan et al. 2003). It thus allows simultaneous substitutions between codons, and it was shown to outperform the previous mechanistic and empirical models in phylogenetic reconstruction (Kosiol et al. 2007). The unrestricted ECM model, which involves a large number of free parameters, was, however, computationally demanding even with fast optimization algorithms (Klosterman et al. 2006).

A reduction in the number of parameters was also achieved by combining three existing empirical amino acid substitution matrices (JJT, mtRev, and cpRev by Jones et al. 1992; Adachi and Hasegawa 1996; Adachi et al. 2000, respectively) with parameters representing the selective pressure and the rate of transition to transversion (Doron-Faigenboim and Pupko 2007). This model was shown to better fit large number of data sets spanning nuclear, viral, mitochondrial, and chloroplast genes than mechanistic and empirical codon models (Doron-Faigenboim and Pupko 2007). This approach has led to the development of several other empirical–mechanistic models based on amino acid propensities (Delport et al. 2010; Rodrigue et al. 2010; De Maio et al. 2013). However, the estimation and interpretation of the parameters borrowed from mechanistic approaches is difficult because the empirical parameters, which represent the known physicochemical attributes or exchangeability rates between codons, already incorporate some aspects of the substitution process (Anisimova and Kosiol 2009).

Overall, models of codon evolution are still lacking generality and the biological relevance of the current models has been questioned (Anisimova and Kosiol 2009). Here, we suggest a new mechanistic model of codon substitution. The model assumes a nucleotide substitution matrix for each nucleotide position in the codon and combines the three nucleotide substitution matrices using a matrix operator to obtain the corresponding codon substitution rate matrix. This fully mechanistic model allows double and triple nucleotide substitutions within a codon without using any representative empirical data set. The performance of our model was assessed by using real and simulated data sets. The biological reality added by our model is important and has profound effects on the fit of the model to diverse data sets tested. It provides a novel direction for further extension and should prove useful for generalizing the estimation of selective forces on molecular data sets.

## New Approaches

### Codon Model Using Kronecker Product

Codons are coded by three consecutive nucleotides that are free to vary and our approach to generalize the model is to assume that substitutions occurring within a codon are independent. The nucleotides present in the three codon positions can therefore change independently and instantaneously. Each nucleotide *i* within a codon is further modeled by a symmetric substitution matrix *q_i_* that allows distinct rates for each type of substitutions. This is in essence similar to the general time-reversible (GTR) substitution matrix (Tavaré 1986), although the state frequencies are not included here (see eq. 3 below):

(1) $$q_{i}=\left(\begin{array}{cccc} 1 & q_{i}^{\text{TC}} & q_{i}^{\text{TA}} & q_{i}^{\text{TG}}\\ q_{i}^{\text{CT}} & 1 & q_{i}^{\text{CA}} & q_{i}^{\text{CG}}\\ q_{i}^{\text{AT}} & q_{i}^{\text{AC}} & 1 & q_{i}^{\text{AG}}\\ q_{i}^{\text{GT}} & q_{i}^{\text{GC}} & q_{i}^{\text{GA}} & 1 \end{array}\right)$$

where the rate of change between nucleotides *j* and *k* in the *i*th codon position is given by $q_{i}^{jk}$ and where $q_{i}^{jk}=q_{i}^{kj}$. Note that each of the three matrices is not normalized at this stage and the 18 parameters (i.e., six per nucleotide positions; see below) are free to vary.

The model of codon evolution (hereafter referred to as KCM) is obtained by combining the three matrices at each codon position using Kronecker product. The result of Kronecker product of the three consecutive 4 × 4 matrices at each codon position is a 64×64 matrix (hereafter referred to as Kronecker matrix), which represents, after some postprocessing described below, the rate transitions between any codons based on the underlying substitution rates of the nucleotides. The initial matrix includes substitutions from and to the three stop codons. We obtained a 61 × 61 Kronecker matrix for sense codons by removing the three rows and columns representing substitutions from and to the three stop codons. The Kronecker matrix is then multiplied by the diagonal matrix of the equilibrium frequencies of codons Π:

(2) $$\Psi =(q_{1}⊗(q_{2}⊗q_{3}))\Pi$$

The substitution matrix that is finally representing how codons are changing through time is as follows:

(3) $$\begin{array}{l} \Psi =\left(\begin{array}{cccc} . & q_{1}^{\text{TC}}q_{\text{2}}^{\text{TC}}q_{\text{3}}^{\text{TC}} & \ldots & q_{\text{1}}^{\text{TG}}q_{\text{2}}^{\text{TG}}q_{\text{3}}^{\text{TG}}\\ q_{\text{1}}^{\text{CT}}q_{\text{2}}^{\text{CT}}q_{\text{3}}^{\text{CT}} & . & \ldots & \vdots \\ \vdots & \ldots & . & q_{\text{1}}^{\text{CG}}q_{\text{2}}^{\text{CG}}q_{\text{3}}^{\text{CG}}\\ q_{\text{1}}^{\text{GT}}q_{\text{2}}^{\text{GT}}q_{\text{3}}^{\text{GT}} & \ldots & \ldots & . \end{array}\right)\\ \times \left(\begin{array}{cccc} \pi_{\text{TTT}} & 0 & \ldots & 0\\ 0 & \pi_{\text{TTC}} & \ldots & 0\\ \vdots & \vdots & \ddots & \vdots \\ 0 & 0 & \ldots & \pi_{\text{GGG}} \end{array}\right) \end{array}$$

where Ψ is a 61 × 61 matrix, $q_{i}^{jk}$ are substitution rates of nucleotide *j* to *k* in the *i*th position of the codon, and $\pi_{m}$ stands for equilibrium frequency of codon *m*. The frequencies $\pi_{m}$ can be any type of codon frequencies and can be estimated empirically from the data as usually done for codon models (Yang 2006).

The KCM model can furthermore be extended to include the effect of selection (Goldman and Yang 1994). For every codon *i* and *j*, the parameter *ω*, which represents the ratio between synonymous and nonsynonymous substitutions, is introduced whenever the transition changes the amino acid coded by the codons, leading to the final matrix *Q*:

(4) $$Q_{ij}=\{\begin{array}{cc} \Psi_{ij}\cdot \omega & \text{ for a nonsynonymous substitution }\\ \Psi_{ij} & \text{ for a synonymous substitution } \end{array}\text{.}$$

Finally, diagonal elements of the *Q* matrix are fixed to ensure that the row sums of *Q* equal zero and *Q* is scaled to obtain an average rate of substitution at equilibrium equals to 1. We therefore count double and triple substitutions as single events, which impose that the branch lengths are measured in expected numbers of substitutions per codon.

### Comparison of Models and Model Selection

The KCM model was first compared using sample corrected Akaike Information Criterion (AICc) (Akaike 1974; Hurvich and Tsai 1989) with the M0 model (Goldman and Yang 1994; Yang 2006) by calculating mean delta AICc values (represented here as AICc_M0_ − AICc_KCM_). The KCM model extends the M0 model by allowing double and triple substitutions and assuming six different substitution rates at each codon position. We do not focus here on the estimation of the selective pressure along sequences, which is averaged over sites and lineages in the M0 model. The main goal is instead to incorporate double and triple substitutions and rate variation among positions in codon models. This has, however, the consequence to introduce some dependency between nucleotide positions in a codon. The effects of this dependency were assessed by restricting the KCM model to include only single substitutions per codon by transforming the *Q* matrix with a binary parameter α∈{0,1} defined as follows:

(5) $${\hat{Q}}_{ij}=\{\begin{array}{cc} Q_{ij} & \text{ for one nucleotide substitution},\\ Q_{ij}\cdot \alpha & \text{ for more than one substitution}. \end{array}$$

With α=0, double and triple substitutions are not allowed and the KCM model is similar to M0 model in this sense. With α=1, the model allows double and triple substitutions and becomes more complex than the M0 model.

One of the main concerns in evaluating a model is the tradeoff between the number of free parameters and goodness of fit (Burnham and Anderson 2002). The main version of KCM includes 19 free parameters (${\text{KCM}}_{19x}$; 6 parameters for each *q_i_* matrix and *ω*), which is more than the parameters of current mechanistic models represented here by M0, which includes 2 parameters (*κ* and *ω*). We investigated the effects of the number of parameters in the KCM model by simplifying the 4 × 4 *q_i_* matrices within the Kronecker framework (eq. 2; table 1). The simplest version, which is referred to as ${\text{KCM}}_{7x}$, has one *q_i_* matrix that is Kronecker multiplied to itself three times. This model further allows only single substitutions per codon by setting α=0 (${\text{KCM}}_{7x\text{M}0}$ model; eq. 5). The ${\text{KCM}}_{7x}$ is still parameter rich in comparison with M0 and has seven free parameters (six that are similar to all *q_i_* matrices and *ω*). Following the same idea, ${\text{KCM}}_{19x}$ can be modified by setting *α* (eq. 5) to 0, which leads to the ${\text{KCM}}_{19x\text{M}0}$ model. We used delta AICc values to compare between the M0 model and the different KCM variants. It should be noted that the codon frequencies, although estimated empirically from the data in all the implementations used here, add between 0 (if frequencies are assumed equal) and 60 parameters to the models. However, within a given type of codon frequencies, the increase in the number of parameters is identical for all the models compared and have therefore no impact when assessing the fit of the different models.

**Table 1.** Different Variants of the KCM Model.

Model Description	Number of Parameters	Fixed Parameter
${\text{KCM}}_{7x\text{M}0}$, $\Gamma =q_{1}^{⊗3}$	7	α=0
${\text{KCM}}_{19x\text{M}0}$, $\Gamma =(q_{1}⊗q_{2})⊗q_{3}$	19	α=0
${\text{KCM}}_{7x}$, $\Gamma =q_{1}^{⊗3}$	7	α=1
${\text{KCM}}_{19x}$ $\Gamma =(q_{1}⊗q_{2})⊗q_{3}$	19	α=1
KCM_19_*_x_*_neutral_ $\Gamma =(q_{1}⊗q_{2})⊗q_{3}$	18	α=1, ω=1

Note.—The symbol *q_i_* refers to the type of substitution matrix at each nucleotide position.

The KCM model was also compared using AICc with the MEC model (Doron-Faigenboim and Pupko 2007), which is a mechanistic–empirical model. To make MEC and KCM models more similar, we assumed one selection ratio for all sites under MEC model, which forces ω=1 and we called it MEC_neutral_. We compared it with KCM under the constraint that ω=1. It was not possible to compare our new models with other approaches, such as the singlet-doublet-triplet (SDT) model (Whelan and Goldman 2004) and the ECM (Kosiol et al. 2007) models, because of difficulties with running the existing implementations of these models.

Finally, we tested whether the nucleotide substitution rates obtained under KCM models were the same as the nucleotide substitution rates obtained under a GTR nucleotide model. For this purpose, we compared the parameters of *q*_1_, *q*_2_, and *q*_3_ obtained under KCM-based models with three GTR model rate matrices estimated from three nucleotide sequences that are generated by concatenating the first, second, and third codon positions. This can be done using the Mgene option of the CodeML software (Yang 2007).

We assessed the performance of the mentioned models on 5 simulated data sets, 3 known proteins, and 100 randomly selected empirical data sets (see Materials and Methods). The first simulated data sets were generated based on M0 model substitution matrix (simulation A), whereas substitution matrices with double and triple rates randomly drawn from normal distributions were added to obtain simulations B, C, and D (see Materials and Methods). We also considered a fifth data set generated by a substitution matrix estimated from the Pandit database (ECM data set; Kosiol et al. 2007). For the empirical data sets, we first considered three known proteins that included the following: 1) β*-Globin* sequences containing 144 codons for 17 vertebrate species extracted from GenBank; 2) *rbcL* sequences from plants containing 447 codons for 20 species; and 3) *pepC* sequences also from plants containing 437 codons for 20 species. We further estimated the different models on 100 empirical data sets randomly selected from the Selectome database (Moretti et al. 2013) whose *ω* values ranged from 0.0054 to 8.66745 as estimated by the M0 model with F3×4 codon frequencies.

The performance of the different models was assessed on all data sets using three types of codon frequencies: F1/16 (all codon frequencies are equal), F3×4 (nucleotide frequencies are estimates of the three codon positions and combined), and *F*61 (codon frequencies are estimated separately for each codon).

## Results

### Simulated Data Sets

The different variants of the KCM model were first compared with the M0 model based on different simulation schemes, which varied based on the level of double and triple substitutions present in the data (no double/triple substitutions for simulation A to approximately 11%, 30%, and 39%, on average, for simulations B, C, and D, respectively). Another set of simulations that we called ECM represented approximately 25% of double and triple substitutions and was created by using a rate matrix derived empirically (Kosiol et al. 2007).

The log likelihood obtained with the F3×4 codon frequencies for the different KCM models under simulation A were, on average, better than the M0 model for all values of the *ω* parameter simulated. However, the mean delta AICc differences for ω=1.0 were −18.71, −20.33, −4.16, and −5.70 for the difference between M0 and ${\text{KCM}}_{19x}$, ${\text{KCM}}_{19x\text{M}0}$, ${\text{KCM}}_{7x}$, and ${\text{KCM}}_{7x\text{M}0}$, respectively, and the ranking of delta AICc did not change qualitatively with different *ω* values (table 2). The better fit of the M0 model in this case is clearly due to the larger number of parameters in each of the KCM variants.

**Table 2.** Mean Delta AICc (standard deviation) Over the 50 Replicates for the Different Simulations Performed.

Simulations	Model	Mean Delta AICc				
		Factor = 0.1	Factor = 0.5	Factor = 1.0	Factor = 2.0	Factor = 10.0
A	${\text{KCM}}_{19x}$	−20.84(6.69)	−20.39(6.38)	−18.71(7.50)	−20.23(6.42)	−17.98(7.84)
	${\text{KCM}}_{7x\text{M}0}$	−20.36(6.33)	−21.77(5.94)	−20.33(6.94)	−21.55(6.05)	−18.66(7.96)
	${\text{KCM}}_{7x}$	−5.77(3.03)	−4.14(4.55)	−4.16(4.81)	−4.35(4.51)	−5.18(3.52)
	${\text{KCM}}_{7x\text{M}0}$	−5.82(3.20)	−5.37(3.57)	−5.70(3.79)	−5.75(3.85)	−6.14(3.32)
B	${\text{KCM}}_{19x}$	3.78(18.35)	40.60(23.19)	44.49(21.63)	55.23(26.96)	65.80(29.51)
	${\text{KCM}}_{7x\text{M}0}$	−22.04(4.84)	−20.22(4.73)	−19.81(5.42)	−19.46(5.70)	−19.97(6.11)
	${\text{KCM}}_{7x}$	18.63(17.77)	55.87(22.88)	59.94(21.80)	70.63(27.70)	79.54(28.93)
	${\text{KCM}}_{7x\text{M}0}$	−8.00(1.74)	−5.71(3.07)	−4.61(3.31)	−4.23(3.26)	−5.43(3.04)
C	${\text{KCM}}_{19x}$	134.83(35.01)	210.45(38.49)	243.46(37.59)	260.84(42.86)	302.18(45.76)
	${\text{KCM}}_{19x\text{M}0}$	−14.50(7.17)	−20.06(4.60)	−20.41(5.54)	−17.94(7.72)	−21.78(6.15)
	${\text{KCM}}_{7x}$	146.46(34.20)	226.87(38.12)	259.05(37.04)	273.08(43.08)	316.93(44.27)
	${\text{KCM}}_{7x\text{M}0}$	−7.32(2.98)	−6.04(2.68)	−5.87(3.72)	−6.13(2.84)	−5.64(2.99)
D	${\text{KCM}}_{19x}$	213.24(52.33)	320.78(57.01)	343.11(46.81)	407.34(55.21)	449.84(53.37)
	${\text{KCM}}_{19x\text{M}0}$	−11.48(32.64)	−14.77(27.79)	−18.55(6.76)	−20.07(7.67)	−21.28(5.73)
	${\text{KCM}}_{7x}$	227.22(53.05)	336.20(57.97)	357.53(46.39)	421.73(55.51)	466.92(52.47)
	${\text{KCM}}_{7x\text{M}0}$	−2.30(30.35)	−2.35(26.98)	−6.80(2.48)	−7.04(2.73)	−5.65(3.76)
ECM	${\text{KCM}}_{19x}$	93.45(17.80)	164.37(31.97)	235.62(43.36)	299.15(42.84)	398.78(35.68)
	${\text{KCM}}_{19x\text{M}0}$	12.19(9.03)	9.31(9.84)	13.79(12.77)	17.14(11.68)	32.55(13.43)
	${\text{KCM}}_{7x}$	98.15(18.31)	162.18(28.51)	229.84(41.43)	287.54(39.80)	373.72(34.05)
	${\text{KCM}}_{7x\text{M}0}$	0.40(4.65)	−0.83(5.35)	−0.38(4.26)	0.34(4.66)	11.74(6.67)

Note.—The analysis is reported for F3×4.The term factor refers to the constant used to multiply the *ω* parameter in the different simulations.

The simulations with no double/triple substitutions resulted in estimated *ω* values that were overall similar to the expected ones (table 3). There is, however, a tendency for large *ω* values to be overestimated and small *ω* values to be underestimated when the model considered double and triple substitutions (i.e., ${\text{KCM}}_{7x}$ and ${\text{KCM}}_{19x}$ for simulation A; table 3). We therefore applied a correction to the *ω* parameter for the ${\text{KCM}}_{19x}$ model because of the variation in rates among nucleotide positions within codons in this model and the allowance of double and triple substitutions (eqs. 6 and 7; see also Goldman and Yang 1994). Although, this correction did not change the pattern observed (corrected *ω* values for ${\text{KCM}}_{19x}$ under simulation A were 0.114, 0.543, 1.137, 2.308, and 14.194, for ω=0.1,0.5,1.0,2.0,10.0, respectively), the variance in *ω* values obtained always included the expected value simulated.

**Table 3.** Mean *ω* (standard deviation) over the 50 Replicates for the Different Simulations Performed.

Simulations	Model	Simulated Parameter				
		Factor = 0.1	Factor = 0.5	Factor = 1.0	Factor = 2.0	Factor = 10.0
A	M0	0.110(0.013)	0.520(0.054)	0.978(0.104)	2.030(0.224)	8.434(1.440)
	${\text{KCM}}_{19x}$	0.114(0.023)	0.543(0.136)	1.137(0.336)	2.308(0.516)	14.194(9.335)
	${\text{KCM}}_{19x\text{M}0}$	0.115(0.023)	0.540(0.133)	1.125(0.332)	2.233(0.469)	10.240(2.918)
	${\text{KCM}}_{7x}$	0.108(0.015)	0.496(0.061)	0.978(0.117)	2.182(0.280)	13.456(8.282)
	${\text{KCM}}_{7x\text{M}0}$	0.110(0.014)	0.512(0.054)	0.980(0.109)	2.042(0.227)	8.458(1.453)
B	M0	0.132(0.014)	0.549(0.066)	1.027(0.137)	1.724(0.407)	4.911(1.710)
	${\text{KCM}}_{19x}$	0.102(0.030)	0.425(0.092)	0.839(0.161)	1.584(0.376)	14.007(15.299)
	${\text{KCM}}_{19x\text{M}0}$	0.108(0.034)	0.426(0.109)	0.811(0.176)	1.393(0.510)	4.228(1.977)
	${\text{KCM}}_{7x}$	0.109(0.013)	0.490(0.069)	0.998(0.144)	1.917(0.432)	20.627(35.725)
	${\text{KCM}}_{7x\text{M}0}$	0.132(0.015)	0.551(0.074)	1.025(0.148)	1.706(0.425)	4.899(1.745)
C	M0	0.185(0.017)	0.607(0.067)	0.986(0.148)	1.407(0.200)	2.524(0.536)
	${\text{KCM}}_{19x}$	0.079(0.020)	0.365(0.071)	0.833(0.286)	1.432(0.467)	10.494(5.773)
	${\text{KCM}}_{19x\text{M}0}$	0.108(0.032)	0.388(0.076)	0.733(0.266)	1.016(0.350)	2.045(0.944)
	${\text{KCM}}_{7x}$	0.096(0.014)	0.448(0.063)	0.948(0.201)	1.765(0.461)	14.205(8.130)
	${\text{KCM}}_{7x\text{M}0}$	0.185(0.018)	0.618(0.076)	1.002(0.152)	1.420(0.230)	2.588(0.524)
D	M0	0.241(0.198)	0.845(1.017)	1.016(0.150)	1.301(0.202)	1.692(0.244)
	${\text{KCM}}_{19x}$	0.078(0.019)	0.405(0.103)	0.744(0.161)	1.632(0.623)	9.559(6.274)
	${\text{KCM}}_{19x\text{M}0}$	0.120(0.037)	0.427(0.103)	0.591(0.168)	0.849(0.257)	1.270(0.629)
	${\text{KCM}}_{7x}$	0.091(0.013)	0.483(0.090)	0.960(0.216)	2.001(0.731)	11.149(7.188)
	${\text{KCM}}_{7x\text{M}0}$	0.209(0.025)	0.706(0.092)	1.036(0.166)	1.334(0.221)	1.687(0.254)
ECM	M0	0.026(0.003)	0.130(0.011)	0.241(0.026)	0.443(0.067)	1.555(0.383)
	${\text{KCM}}_{19x}$	0.006(0.003)	0.034(0.008)	0.066(0.015)	0.122(0.022)	0.633(0.163)
	${\text{KCM}}_{19x\text{M}0}$	0.009(0.005)	0.052(0.014)	0.102(0.031)	0.192(0.044)	0.698(0.240)
	${\text{KCM}}_{7x}$	0.008(0.002)	0.053(0.006)	0.099(0.016)	0.194(0.026)	1.108(0.296)
	${\text{KCM}}_{7x\text{M}0}$	0.026(0.003)	0.123(0.011)	0.226(0.025)	0.427(0.064)	1.460(0.359)

Note.—The values given for the ${\text{KCM}}_{19x}$ and ${\text{KCM}}_{7x}$ models are the uncorrected ones (see text). The analysis is reported for F3×4. The term factor refers to the constant used to multiply the *ω* parameter in the different simulations.

**Table 4.** Estimated Log Likelihood (ln *L*), AICc, and *ω* Parameters (corrected for the ${\text{KCM}}_{19x}$ model) for Vertebrate *β-Globin* and the Plants *rbcL* and *pepC* Genes.

Models	*β-Globin*			*rbcL*20			*pepC*20		
	−ln *L*	AICc	Ω	−ln *L*	AICc	*ω*	−ln *L*	AICc	*ω*
M0	3,815.5	7,635.1	0.23685	4,362.7	8,729.4	0.10116	9,783.4	19,571	0.06597
${\text{KCM}}_{7x\text{M}0}$	3,799.9	7,614.1	0.20640	4,336.70	8,687.5	0.08671	9,734.95	19,484	0.06093
${\text{KCM}}_{19x\text{M}0}$	3,710.5	7,460.8	0.1409	4,301.86	8,642.3	0.0832	9,595.99	19,231	0.0443
${\text{KCM}}_{7x}$	3,694.78	7,403.8	0.11417	4,297.31	8,608.7	0.06580	9,541.91	19,098	0.04428
${\text{KCM}}_{19x}$	3,601.16	7,242.2	0.0706	4,263.39	8,565.4	0.0652	9,367.98	18,775	0.0291
MEC_neutral_	3,840.4	7,697.9	1.129	4,557.3	9,130.9	1.189	10,504.9	21,026.9	1.139
KCM_19_*_x_*_neutral_	3,659.9	7,361.3	1.000	4,328.5	8,693.5	1.000	9,649.0	19,335.0	1.000

The pattern obtained is very different once some amount of double and triple substitutions are introduced and drastic increase in log-likelihood values were observed, reflected in the mean delta AICc, for the variants of the KCM model (simulations B–D and ECM; table 2). This was irrespective of the amount of double and triple substitutions present in the data. The different KCM variants clearly outperformed the M0 model with a difference in mean delta AICc for ${\text{KCM}}_{19x}$ and ${\text{KCM}}_{7x}$ between 214.367 and 223.405 (simulations B–D and ECM; table 2). Forcing the KCM models to ignore any substitutions implying double and triple substitutions (i.e., ${\text{KCM}}_{19x\text{M0}}$ and ${\text{KCM}}_{7x\text{M}0}$) resulted in log likelihood that were comparable with the M0 model (simulations B–D and ECM; table 2) and mean delta AICc between −9.868 and −3.5925 (simulations B–D and ECM; table 2).

The large differences seen when comparing, on one hand, ${\text{KCM}}_{19x}$ with ${\text{KCM}}_{19x\text{M0}}$ and, on the other hand, ${\text{KCM}}_{7x}$ with ${\text{KCM}}_{19x}$${\text{KCM}}_{7x\text{M}0}$ indicate that the number of parameters in itself was not responsible for the large improvement of the delta AICc observed with models allowing double and triple substitutions.

**Fig. 1.**
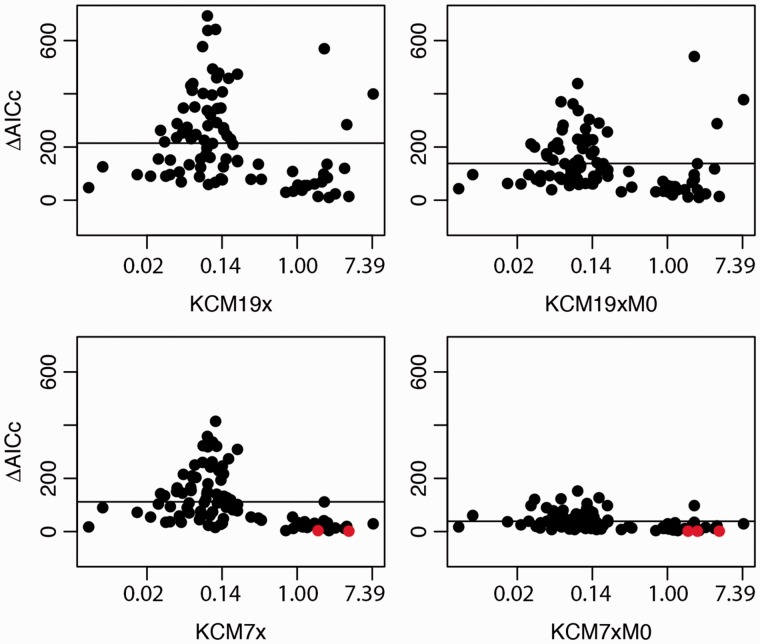
Delta AICc plots comparing the performance of the M0 model with KCM models (${\text{KCM}}_{7x}$, ${\text{KCM}}_{7x\text{M}0}$, ${\text{KCM}}_{19x}$, ${\text{KCM}}_{19x\text{M}0}$) on 100 empirical data sets randomly selected from Selectome database. For each empirical data set, we evaluated the maximum-likelihood value of the M0 model and the KCM variants and compared the delta AICc to penalize the 2 free parameters of the M0 model and the 7, 7, 19, and 19 free parameters of the KCM models, respectively. For each plot, a black horizontal line is drawn for the mean delta AICc value of the empirical data set. The codon frequencies used were the products of the observed nucleotide frequencies at each of the three codon positions (F3×4; Yang and Bielawski 2000). Empirical data sets with delta AICc < 4 are shown in red.

Changing the type of codon frequencies from F3×4 to F1/61 had a drastic effect on the fit of the models when we analyzed the simulations with low amounts of double and triple substitutions (simulation A; supplementary fig. S1, Supplementary Material online). Under equal codon frequencies, the mean delta AICc of ${\text{KCM}}_{19x}$ became much better than M0 (from −18.71 to 2.07 for ω=1), whereas the mean delta AICc of ${\text{KCM}}_{7x}$ changed from −4.16 to −2.75, again for ω=1, even though this data set was simulated based on the substitution matrix of M0 itself. This result suggests that the M0 and ${\text{KCM}}_{7x}$ models, which do not allow rate variability within codon positions, depend heavily on the codon frequencies to compensate the model restrictions. When the amount of double and triple substitutions increases further (simulations B, C, D, and ECM), the fit of the KCM variants is much higher than M0 regardless of the frequency mode. This suggests that the ability of the KCM models to incorporate double and triple substitutions becomes much more relevant than the rate variation within codons (fig. 1 and supplementary figs. S1 and S2, Supplementary Material online). The results for the *F*61 codon frequencies are similar to those for F3×4 and are therefore not shown.

The estimated *ω* parameters under the simulation schemes B–D and ECM were correctly estimated by ${\text{KCM}}_{19x}$ and ${\text{KCM}}_{7x}$ models, whereas a strong bias was introduced when the models did not allow double and triple substitutions (table 4). This bias is either an overestimation, when the data are simulated under purifying selection (ω=0.1,0.5) or a strong underestimation in the presence of positive selection (ω=2.0,10.0). Although the exact *ω* value for the ECM simulations is not known in advance (Kosiol et al. 2007), we observed that the *ω* value estimated by the ${\text{KCM}}_{19x}$ and ${\text{KCM}}_{7x}$ models increased linearly with the factor that we applied to *ω*, whereas the M0 model was not sensitive to this factorization of the *ω* parameter (table 4).

The model comparisons showed that ${\text{KCM}}_{19x}$ is able to outperform standard models of codon evolution. However, these evaluations do not inform about the accuracy of the parameters estimated by our new model and their biological relevance. This was assessed by simulating data sets under either the M0 or ECM models and comparing the rates of substitutions of codons obtained with the M0, ${\text{KCM}}_{19x}$, and ECM models. The distribution of the median values over the 50 simulations for each entity of the 61×61 rate matrix showed that ${\text{KCM}}_{19x}$ substitution rates can approximate either the M0 (supplementary fig. S3, Supplementary Material online) or the ECM (supplementary fig. S4, Supplementary Material online) model. In contrast, ECM and M0 are not able to represent as well the substitution rates of M0 and ECM, respectively. Indeed, the Euclidian distances measured on the entities of the 61×61 matrix were smaller for ${\text{KCM}}_{19x}$ than for any of the other alternative models (i.e., 1.005 for ${\text{KCM}}_{19x}$ versus 2.281 for *ECM* on simulations based on the M0 model; 1.812 for ${\text{KCM}}_{19x}$ versus 2.789 for M0 on simulations based on the ECM model). Moreover, the branch lengths estimated by ${\text{KCM}}_{19x}$ are correlated with the ones obtained by the model used for the simulations (0.69 with M0 and 0.90 for ECM).

For computational simplicity, we simulated data sets that contained only 150 codons alignments. This could have an impact on the estimation of the parameters of the models and we therefore checked for simulations A, B, and ECM if our estimation of the *ω* parameter (table 4) was comparable with simulations done on 3,500 codons. The median of the *ω* was the same for both alignment lengths and a reduction in the variance of the parameter estimates was observed with larger alignments (supplementary table S1, Supplementary Material online). The use of 150 codon alignments should, therefore, not affect our main conclusions.

### Empirical Data Sets

The performance of the KCM model and its extensions on the three empirical data sets are shown in table 4. In each case, the best model, as measured with the AICc criteria, was ${\text{KCM}}_{19x}$, which allows both a different substitution matrix per codon position and double and triple substitutions to occur (table 1). Overall, the KCM variants outperformed the M0 model, when allowing selective pressure to be estimated, or MEC_neutral_ when *ω* was fixed to 1 in KCM (table 4).

For the *β-globin* gene, the AICc for the ${\text{KCM}}_{7x\text{M}0}$ model was lower than the M0 model by 21.0. Extending the ${\text{KCM}}_{7x\text{M}0}$ model to allow variable substitution rates among nucleotide positions within a codon (i.e., ${\text{KCM}}_{19x\text{M}0}$) further improved the AICc by 153.30, whereas allowing double and triple substitutions (i.e., ${\text{KCM}}_{7x}$) resulted in a reduction in AICc of 210.30 (table 4). Combining these two aspects into the more general ${\text{KCM}}_{19x}$ model clearly gave the best AICc value for the *β-globin* data sets and resulted in a reduction of ${\text{KCM}}_{7x\text{M}0}$ AICc by 371.90. The same trend was observed for the two plant data sets tested here (table 4).

The *β-globin* gene was also used to compare the three GTR matrices estimated by partitioning the sequences into independent codon positions (Mgene option in CodeML) and the *q_i_* matrices obtained from the ${\text{KCM}}_{7x\text{M}0}$ and ${\text{KCM}}_{19x}$ models. The substitution rates estimated by ${\text{KCM}}_{19x\text{M}0}$ were very similar to those estimated for each codon positions and the mean relative error per matrix entities was equal to 5.8% with no obvious distinction between codon positions. Allowing double and triple substitutions by using the ${\text{KCM}}_{19x}$ model changed the pattern, and the mean relative error per entities increased to 22.6%. The largest discrepancies observed between the ${\text{KCM}}_{19x}$ *q_i_* matrices and the three independent GTR matrices were found at the first position, followed by the third codon positions (data not shown), whereas the values of the rate matrix for the second position were highly similar between the two models.

The importance of incorporating double and triple substitutions can be further understood by looking at the substitution rates found in each 61 × 61 matrix. Double and triple substitution rates estimated under ${\text{KCM}}_{19x}$ ranged from <0.001 to 0.145 for *β-globin*, from <0.001 to 0.121 for *rbcL*, and from <0.001 to 0.272 for *pepC*. A graphical representation of the rate matrices estimated by ${\text{KCM}}_{19x}$ for *β-globin* is shown in the supplementary fig. S5, Supplementary Material online. For each empirical data sets, the number of double and triple substitutions with rates >0.001 is high (44.2%, 28.3%, and 40.0% for *β-globin*, *rbcL*, and *pepC*, respectively), and there are several double and triple substitutions with higher substitution rates than single substitutions (supplementary fig. S5, Supplementary Material online).

In contrast to the simulated data sets, the estimated ratio of nonsynonymous versus synonymous substitutions (*ω*) was smaller under the different KCM models than with M0 for all empirical data sets tested. The incorporation of more complexity in the KCM variants lead to a sharp reduction in the estimated *ω* values for each data sets tested (table 4). Allowing both rate variation among codon positions and multiple nucleotide substitutions per codons (i.e., ${\text{KCM}}_{19x}$; table 4) reduced the estimated *ω* by half or more in all empirical data sets. This is similar to what was observed for the simulations B–D under low *ω* values (table 4) and would suggest that double and triple substitutions are present in those empirical data sets.

The M0 model is certainly making strong assumptions that might not be relevant for the empirical data tested here and the improvements seen with the KCM variants might therefore be expected. The MEC model is taking another route to achieve biological realism, but our results show that the KCM_19_*_x_*_neutral_ model is also fitting the different empirical data sets much better (table 4). For instance, the reduction in AICc obtained with KCM_19_*_x_*_neutral_ on the *β-globin* gene reached 336.6 over the MEC_neutral_ model (table 4). Again, similar reductions in AICc were observed for the two plant data sets (table 4).

The Almost Invariant Sites (AIS) analyses on the two plant data sets illustrate the biological consequences of the substitution matrix of codons obtained by the KCM model. The creation of 20 codon classes resulted in a clear assemblage of codons into their amino acids and a high similarity between the clusters was found by analyzing either ${\text{KCM}}_{19x}$ or M0 substitution matrix (table 5). This was particularly true for the *rbcL* data set, whereas the *pepC* data set showed slight differences (e.g., amino acids phenylalanine [F] and isoleucine [I] are grouped together with KCM but not with M0; table 5). Similar results were obtained when the partitioning was done based on the seven physicochemical properties of amino acids (table 6).

**Table 5.** Partitions of the Codons into 20 Categories Based on the Substitution Rate Matrix of 2 Genes Obtained Under ${\text{KCM}}_{19x}$ and M0 Models Using AIS Algorithm.

	AIS Analysis	
Gene	KCM	M0
Sets for *rbcL*	{ F (TTT) F (TTC) }	{ F (TTT) F (TTC) }
	{ S (TCT) S (TCC) S (TCA) S (TCG) A (GCT) A (GCC) A (GCA) A (GCG) }	{ S (TCT) S (TCC) S (TCA) S (TCG) A (GCT) A (GCC) A (GCA) A (GCG) }
	{ Y (TAT) Y (TAC) }	{ Y (TAT) Y (TAC) }
	{ C (TGT) C (TGC) }	{ C (TGT) C (TGC) }
	{ W (TGG) }	{ W (TGG) }
	{ L (TTA) L (TTG) L (CTT) L (CTC) L (CTA) L (CTG) }	{ L (TTA) L (TTG) L (CTT) L (CTC) L (CTA) L (CTG) }
	{ P (CCT) P (CCC) P (CCA) P (CCG) }	{ P (CCT) P (CCC) P (CCA) P (CCG) }
	{ H (CAT) H (CAC) }	{ H (CAT) H (CAC) }
	{ Q (CAA) Q (CAG) }	{ Q (CAA) Q (CAG) }
	{ R (CGT) R (CGC) R (CGG) }	{ R (CGT) R (CGC) R (CGG) }
	{ R (CGA) R (AGA) R (AGG) }	{ R (CGA) R (AGA) R (AGG) }
	{ I (ATT) I (ATC) I (ATA) T (ACT) }	{ I (ATT) I (ATC) I (ATA) T (ACT) }
	{ M (ATG) }	{ M (ATG) }
	{ T (ACC) T (ACA) T (ACG) }	{ T (ACC) T (ACA) T (ACG) }
	{ N (AAT) N (AAC) D (GAT) D (GAC) }	{ N (AAT) N (AAC) D (GAT) D (GAC) }
	{ K (AAA) K (AAG) }	{ K (AAA)K (AAG) }
	{ S (AGT) S (AGC) }	{ S (AGT)S (AGC) }
	{ V (GTT) V (GTC) V (GTA) V (GTG) }	{ V (GTT) V (GTC) V (GTA) V (GTG) }
	{ E (GAA) E (GAG) }	{ E (GAA) E (GAG) }
	{ G (GGT) G (GGC) G (GGA) G(GGG) }	{ G (GGT) G (GGC) G (GGA) G (GGG) }

sets for *pepC*	{ F(TTT) F(TTC) I(ATT) I(ATC) I(ATA) }	{ F(TTT) F(TTC) }
	L(TTA) L(TTG) L(CTT) L(CTC) L(CTA) L(CTG) }	{ L(TTA) }
	{ S(TCT) S(TCC) S(TCA) S(TCG) }	{ L(TTG) L(CTT) L(CTC) L(CTA) L(CTG) R(CGC) R(CGG) }
	{ Y(TAT) Y(TAC) }	{ S(TCT) S(TCC) S(TCA) S(TCG) }
	{ C(TGT) }	{ Y(TAT) Y(TAC) }
	{ C(TGC) }	{ C(TGT) }
	{ W(TGG) }	{ C(TGC) }
	{ P(CCT) P(CCC) P(CCA) P(CCG) }	{ W(TGG) }
	{ H(CAT) H(CAC) }	{ P(CCT) P(CCC) P(CCA) P(CCG) }
	{ Q(CAA) Q(CAG) }	{ H(CAT) H(CAC) D(GAT) D(GAC) }
	{ R(CGT) R(CGC) R(CGA) R(CGG) R(AGA) R(AGG) }	{ Q(CAA) Q(CAG) }
	{ M(ATG) }	{ R(CGT) R(CGA) R(AGA) R(AGG) }
	{ T(ACT) T(ACC) T(ACA) T(ACG) }	{ I(ATT) I(ATC) I(ATA) V(GTT) V(GTC) V(GTA) V(GTG) }
	{ N(AAT) N(AAC) }	{ M(ATG) }
	{ K(AAA) K(AAG) E(GAA) E(GAG) }	{ T(ACT) T(ACC) T(ACA) T(ACG) }
	{ S(AGT) S(AGC) G(GGA) G(GGG) }	{ N(AAT) N(AAC) }
	{ V(GTT) V(GTC) V(GTA) V(GTG) }	{ K(AAA) K(AAG) E(GAA) E(GAG) }
	{ A(GCT) A(GCC) A(GCA) A(GCG) }	{ S(AGT) S(AGC) }
	{ D(GAT) D(GAC) }	{ A(GCT) A(GCC) A(GCA) A(GCG) }
	{ G(GGT) G(GGC) }	{ G(GGT) G(GGC) G(GGA) G(GGG) }

**Table 6.** Partitions of Codons into Seven Categories Based on Substitution Rate Matrix of Two Genes Obtained Under ${\text{KCM}}_{19x}$ and M0 Models Using AIS Algorithm.

Genes	AIS Analysis	
	KCM	M0
Sets for *rbcL*	{F (TTT) F (TTC) L (TTA)L (TTG)S (TCT)S (TCC)S (TCA)S (TCG)L (CTT)L (CTC)L (CTA)L (CTG) P (CCT) P (CCC)P (CCA)P (CCG)I (ATA)V (GTT)V (GTC)V (GTA)V (GTG)}	{F (TTT) F (TTC) L (TTA)L (TTG)S (TCT)S (TCC)S (TCA)S (TCG)L (CTT)L (CTC)L (CTA)L (CTG) N (AAT)N (AAC)K (AAG)S (AGT)R (AGA)D (GAT)D (GAC)E (GAA)E (GAG)G (GGT)G (GGC)G (GGA)G (GGG)}
	{Y (TAT)Y (TAC)H (CAT)H (CAC)N (AAT)N (AAC)D (GAT)D (GAC)}	{Y (TAT)Y (TAC)C (TGT)C (TGC)H (CAT)H (CAC)S (AGC)}
	{C (TGT)C (TGC)W (TGG)S (AGT)S (AGC)G (GGT)G (GGC)G (GGA)G (GGG)}	{W (TGG)V (GTG)}
	{Q (CAA)Q (CAG)K (AAA)K (AAG)}	{Q (CAA)Q (CAG)R (CGA)R (CGG)K (AAA)R (AGG)}
	{R (CGT)R (CGC)R (CGA)R (CGG)T (ACT) T (ACC)T (ACA)T (ACG)R (AGA)R (AGG)A (GCT) A (GCC)A (GCA)A (GCG)}	{P (CCT) P (CCC)P (CCA)P (CCG)R (CGT)R (CGC)}
	{I (ATT)I (ATC)M (ATG)}	{I (ATT)I (ATC)I (ATA)M (ATG)}
	{E (GAA)E (GAG)}	{T (ACT) T (ACC)T (ACA)T (ACG)V (GTT)V (GTC)V (GTA)A (GCT) A (GCC)A (GCA)A (GCG)}

Sets for *pepC*	{F (TTT) F (TTC) L (TTA)S (TCT)S (TCC)S (TCA)S (TCG)I (ATT)I (ATC)I (ATA)T (ACT) T (ACC)T (ACA)T (ACG)V (GTT)V (GTC)V (GTA)V (GTG)}	{F (TTT) F (TTC) L (TTA)L (TTG)L (CTT)L (CTC)L (CTA)L (CTG) H (CAT)H (CAC)R (CGC)R (CGA)R (CGG)M (ATG) V (GTA)V (GTG)}
	{L (TTG)L (CTT)L (CTC)L (CTA)L (CTG) P (CCT) P (CCC)P (CCA)P (CCG)M (ATG)}	{S (TCT)S (TCC)S (TCA)S (TCG)P (CCT) P (CCC)P (CCA)P (CCG)T (ACT) T (ACC)T (ACA)T (ACG)V (GTT)V (GTC)A (GCT) A (GCC)A (GCA)A (GCG)}
	{Y (TAT)Y (TAC)C (TGT)C (TGC)S (AGT)S (AGC)}	{Y (TAT)Y (TAC)C (TGC)N (AAT)N (AAC)D (GAT)D (GAC)E (GAA)E (GAG)G (GGC)G (GGG)}
	{W (TGG)R (CGT)R (CGC)R (CGA)R (CGG)R (AGA)R (AGG)G (GGA)G (GGG)}	{C (TGT)R (CGT)S (AGC)G (GGT)G (GGA)}
	{H (CAT)H (CAC)Q (CAA)Q (CAG)N (AAT)N (AAC)K (AAA)K (AAG)D (GAT)D (GAC)E (GAA)E (GAG)}	{I (ATT)I (ATC)I (ATA)}
	{A (GCT) A (GCC)A (GCA)A (GCG)}	{W (TGG)S (AGT)}
	{G (GGT)G (GGC)}	{Q (CAA)Q (CAG)K (AAA)K (AAG)R (AGA)R (AGG)}

Finally, we compared the KCM and M0 models on 100 empirical data sets randomly selected from the Selectome database (Moretti et al. 2013). We showed that KCM variants outperform the other models in all but two data sets (fig. 1 and supplementary fig. S6, Supplementary Material online). Furthermore, the estimation of the average rate of transitions and transversion by ${\text{KCM}}_{19x}$ and ${\text{KCM}}_{7x}$was very close to the M0 model (supplementary fig. S7, Supplementary Material online), which suggests that KCM models can capture biologically relevant aspects of the evolution of these protein-coding genes. For F1/61 codon frequencies, the mean value of delta AICc for the 100 empirical data sets evaluated with the ${\text{KCM}}_{19x}$ model was 151.98, whereas it was 53.05 for ${\text{KCM}}_{7x}$ (supplementary fig. S8, Supplementary Material online). These values changed when we used the F3×4 codon frequencies with mean delta AICc increasing to 214.41 and 111.84 for ${\text{KCM}}_{19x}$ and ${\text{KCM}}_{7x}$, respectively (fig. 1). The results for the *F*61 codon frequencies are similar to those for F3×4 and are not shown.

## Discussion

We proposed a new model of codon evolution that incorporates rate variation within codon positions and allows double and triple substitutions between codons. It represents an attempt to capture the real processes behind protein-coding sequence evolution and generalizes the current mechanistic models without relying on any empirical data.

A fully parametric model of codon substitution defined by a 61 × 61 rate matrix would require the estimation of 1,830 parameters if the model is assumed symmetrical. The KCM model described here reduces this parameter space through the use of the Kronecker product of three consecutive 4 × 4 nucleotide substitution matrices, that is, *q*_1_, *q*_2_, and *q*_3_ (eq. 3). This allows the substitution process to be modeled by only 18 rate parameters and one selection parameter *ω*. The parameters of the *q_i_* matrices represent the contribution of the corresponding nucleotide substitutions to the evolution of codons. However, the resulting substitution rates of codons are not simply multiplications of substitution rates of the three corresponding nucleotide substitution rate matrices as would be done by assuming that the three positions evolved independently. Dependency between codon positions is well-known (Robinson et al. 2003; Whelan 2008), and our model incorporates some of this dependency during the building of the codon substitution matrix through the Kronecker product. In contrast to context-dependent models (Baele et al. 2011) or to the use of codon partitions (like the option Mgene in CodeML), our approach maintains the codon as the unit of evolution and, in addition to nucleotide substitution parameters, equilibrium frequency of codons, that is, *F*61, F3×4 model, and the branch lengths play roles in estimating the substitution rates of codons. However, our model cannot incorporate the effect of neighboring bases on the substitution process within codons (Fedorov et al. 2002; Morton and Wright 2007).

The KCM-based models explained the three real data sets better than the M0 model considered in this article. Our results highlight that allowance of double and triple nucleotide substitutions in a codon at each time interval is an important aspect to model the evolutionary process underlying protein-coding sequence data. This is in line with a recent study that showed that the most relevant parameters for codon models are *ω* and double and triple substitutions (Zoller and Schneider 2010). On the other hand, the KCM_19xNeutral_ model explained the empirical data sets better than the MEC_neutral_ model, even though the MEC model uses empirical data and also allows double and triple substitution. This suggests that rate variation among nucleotides within a codon, which is a component present in our KCM model but not incorporated explicitly in the MEC model, is another relevant aspect to consider beside those proposed by Zoller and Schneider (2010). Our results show the utility to generalize codon models of substitution and suggest that the KCM-based models that we propose are able to incorporate the most important factors acting on the evolution of protein-coding genes (tables 2 and 4).

Our KCM models, in particular the ${\text{KCM}}_{19x}$, are more parameter rich than M0 and MEC_neutral_ models. Overparameterization can therefore be considered as a possible explanation for the better fit observed in our results. We have chosen AICc measures in our model comparisons to reduce the risk that the decrease in likelihood of the KCM models could be the result of a larger number of parameters. Simulation A gives some insight into the effect of overparameterization in our models. Because the data were simulated under the M0 model, all codon positions have the same substitution rate matrix and no differentiation between nucleotide positions is introduced. In this case, one would expect the delta AICc values for the ${\text{KCM}}_{19x}$ model to be lower than or at least very similar to those for ${\text{KCM}}_{7x}$.

The different variants of the KCM models allow, however, a better exploration of the effects of the increased number of parameters. In particular, comparisons between the ${\text{KCM}}_{19x}$ or ${\text{KCM}}_{7x}$ and their respective variants allowing only single substitutions (i.e., ${\text{KCM}}_{19x\text{M}0}$ and ${\text{KCM}}_{7x\text{M}0}$, respectively), which have the same number of parameters, suggest that the effect of the number of parameters, although present, is minimized (tables 2 and 4). The changes in likelihood values are due to the allowance of double and triple substitutions in the substitution process. Our results concur with other studies (Whelan and Goldman 2004; Doron-Faigenboim and Pupko 2007; Kosiol et al. 2007) and reinforce the need to better explore the effects of biological realism in models of codon evolution.

Although different KCM variants obtained better AICc values when compared with other models, it is important to understand whether the codon substitution rate matrix obtained by our models is biologically meaningful and represents the substitution patterns expected between amino acids. This was checked by first extracting the codon substitution rate matrix obtained under the ${\text{KCM}}_{19x}$ model for the *β-globin* gene and generating a schematic representation of the matrix (supplementary fig. S5, Supplementary Material online). It had a very similar configuration than the published representations (Kosiol et al. 2007; De Maio et al. 2013), which suggest that the distribution of codon substitutions in the ${\text{KCM}}_{19x}$ model is biologically plausible. Second, the biological relevance of the codon substitutions was assessed by the AIS software (Lio and Goldman 1998) applied, again, to the ${\text{KCM}}_{19x}$ codon substitution rate matrix obtained from the two plant data sets (tables 5 and 6). The small variations observed between real codon sets and partitions obtained under the ${\text{KCM}}_{19x}$ model was also seen under the M0 model. This suggests that model assumptions are not causing these deviations, but that it is either due to the nature of the data sets or to some common assumptions of mechanistic models of codon evolution. Moreover, by comparing KCM substitution rates with standard models when the data are simulated under M0 and ECM models, we showed that KCM not only outperforms standard models but that it also correctly estimates the parameter values.

Beside the ability to account for double and triple substitutions, the ${\text{KCM}}_{19x}$ variant of our model can also capture the rate variability existing between the three positions within a codon. There has been several attempts to incorporate the variability among sites in codon models (Pond and Muse 2005; Mayrose et al. 2007; Rubinstein et al. 2011) and to allow both synonymous and nonsynonymous rates to vary. However, only empirical models that estimate exchangeability rates from existing data have been extending this variability to different positions within a codon (Kosiol et al. 2007). Here, we provide further evidence that this variation might be important and that an accurate modeling of protein evolution should go beyond the simple consideration of synonymous versus nonsynonymous changes. For example, the investigation of multilayered selective pressure (Rubinstein et al. 2011) that model the level of selection at the protein and DNA/RNA levels is of interest in this context.

In our results, the difference between AICc values of M0 and ${\text{KCM}}_{7x}$ captures the model ability to take into account the double and triple substitutions. The difference between the AICc values of ${\text{KCM}}_{7x}$ and ${\text{KCM}}_{19x}$, that both incorporates double and triple substitutions, shows the model ability to take into account the variability within the codon positions. The advantage of the ${\text{KCM}}_{19x}$ is already evident in the ECM simulations (table 2), but this effect is increased drastically for empirical data sets (fig. 1). The better performance of ${\text{KCM}}_{19x}$ over the restricted model ${\text{KCM}}_{7x}$ is observable for all types of codon frequencies and especially with F3×4 in the ECM simulations and empirical data sets, which show an increased variability between the rate matrices of the three codon positions over the simulations A–D (between 10% and 30% more difference observed; data not shown). This has already been shown in other empirical data sets (Bofkin and Goldman 2007) and highlights the importance of correctly estimating the codon frequencies in such models (Aris-Brosou and Bielawski 2006). This result further suggests that the rate variability within codons captured by ${\text{KCM}}_{19x}$ is modeling important aspects of protein-coding gene evolution.

The variability within codon positions is in part associated with the codon usage bias. For example, the difference between the AICc values of ${\text{KCM}}_{19x}$ and ${\text{KCM}}_{7x}$ were highly dependent on the type of codon frequencies used (supplementary fig. S8, Supplementary Material online). This indicates that ${\text{KCM}}_{19x}$ can incorporate codon usage bias through the three different rate matrices used to build this model. Therefore, this ability can rescue the model even when we assumed that the codon frequencies were equal. However, the better fit of ${\text{KCM}}_{19x}$ remains even under the F3×4 type of codon frequencies, which indicates that other factors are affecting the amount of rate variability observed within codon positions (fig. 1). One of these factors could be selective pressure, which will push for substitution rates to differ between first, second, and third positions (Nei and Gojobori 1986; Goldman and Yang 1994). It would be important to further study this aspect to better understand the process behind the evolution of protein-coding evolution, but this is beyond the scope of this study.

One of the advantages of mechanistic models is that their parameters are defined based on biological processes (Yang 2006) and allow a direct test of the relevance of these parameters through AIC or likelihood-based measures. In the case of KCM models, the parameters of the *q_i_* matrices are borrowed from the GTR model of nucleotide substitution (Tavaré 1986). However, we estimate them from codon data and not directly from nucleotide data and the biological meaning of the parameters included in *q_i_* is not straightforward. The other approach to incorporate double and triple substitutions in a mechanistic codon model (Whelan and Goldman 2004) did not have the same interdependency between codon positions that the Kronecker product introduces. Their rate matrices were therefore formed by rescaled single, double, and triple instantaneous substitution rates that are comparable with the events occurring in nucleotide model (Whelan and Goldman 2004). The effect of the interdependency in the KCM model is readily seen in the comparison between the partitioned codon data analyzed with the Mgene option of CodeML and the full estimation under the ${\text{KCM}}_{19x}{}_{\text{M0}}$ and ${\text{KCM}}_{19x}$ models. The parameters of the former model were very similar to the parameters obtained in the three GTR substitution matrices, whereas the deviation increase to about 22% with the latter especially in the matrices defining the first and third codon positions. This suggests that the parameters of the *q* matrices in ${\text{KCM}}_{19x}$ are nucleotide substitution parameters in protein-coding area under codon constraints, which can be compared with the exchangeability rates or replacement probabilities used in empirical codon models (Doron-Faigenboim and Pupko 2007; Kosiol et al. 2007).

The mechanistic codon model that we present here has 19 free parameters, which are combined to represent the full set of codon transition rates. It can therefore consider single, double, and triple nucleotide substitutions per codon within a small interval of time and allows the modeling of rate variation between codon positions. The codon transition rates estimated by ${\text{KCM}}_{19x}$ are biologically relevant and our new model can have a better fit than current models on simulated and empirical data sets. However, the estimation of *ω* by KCM models slightly differ from the M0 and ECM models. The estimation of the selective pressure when double and triple substitutions are incorporated is more complex (Kosiol et al. 2007; De Maio et al. 2013) and applying existing correction to the estimated *ω* is not yet satisfying. Further investigations should be done to clarify the relationship between the *ω* value estimated by ${\text{KCM}}_{19x}$ and the *ω* values of the standard model (M0) and empirical models (ECM).

## Conclusion

The KCM model is an attempt to generalize mechanistic models of codon evolution. It uses a mathematical operator, the Kronecker product, to increase the number of effective parameters, which allows the inclusion of double and triple nucleotide substitutions. We show that the KCM model can lead to improvements of the likelihood when compared with traditional models of codon evolution. This has consequences on key parameters used to describe the evolution of protein-coding sequences. In particular, our simulations suggest that the effect of double and triple substitutions can be important for the identification of selective pressure. It is evident that assuming a single *ω* value for all sites and branches has now been shown to be unrealistic (Zhang et al. 2005; Anisimova and Kosiol 2009); we can suppose that the biases that we observed when data with double and triple substitutions are analyzed under the M0 model will be maintained. This is clearly calling for further studies to understand the potential extent of such bias and the extension of the KCM model that we proposed could represent one possibility.

## Materials and Methods

### Correcting Synonymous and Nonsynonymous Ratio

The rate variation across nucleotide sites in a protein-coding region is usually assumed to be due to selection pressure, and this is modeled by the *ω* parameter (Yang 2006). However, the KCM model allows for rate variation across nucleotide positions in a codon by considering a separate GTR matrix for each nucleotide position. The estimation of *ω* as described in equation (4) is therefore biased and a correction has to be applied. To do so, we first estimate the synonymous substitution rate per codon (Goldman and Yang 1994)

(6) $$\rho_{\text{s}}=\sum_{i=1}^{61}\sum_{j=1,j\neq i,{\text{aa}}_{i}={\text{aa}}_{j}}^{61}\pi_{i}Q_{ij}\text{.}$$

Similarly, the nonsynonymous rate per codon $\rho_{a}$ can be calculated by summing $\pi_{i}Q_{ij}$ over all codons *i*, *j* coding for different amino acids. Then, the synonymous and nonsynonymous rate per codon parameters $\rho_{s}^{\infty }$ and $\rho_{a}^{\infty }$ are estimated under neutral selection (Nei and Gojobori 1986; Goldman and Yang 1994) by using the same nucleotide substitution matrix for the three codon positions. This is obtained by setting ω=1 and averaging the three nucleotide substitution matrices used to estimate $\rho_{\text{s}}$ and $\rho_{\text{a}}$. Then,

(7) $$\omega_{\text{KCM}}=K_{\text{a}}/K_{\text{s}}=\rho_{\text{a}}\rho_{\text{s}}^{\infty }/\rho_{\text{s}}\rho_{\text{a}}^{\infty }$$

where $\omega_{KCM}$ is the corrected *ω* for the KCM model, *K*_s_ the number of synonymous substitutions per synonymous site, and *K*_a_ the number of nonsynonymous substitutions per nonsynonymous site.

### Simulated and Empirical Data Sets

The performances of the KCM models were assessed using both simulated and empirical data sets. We created five simulated data sets to assess the effects of the amount of double and triple substitutions on the estimation of the *ω* parameter and the likelihood of the model. The first simulated data set, A, is not composed of any double and triple substitutions and was created by using the settings of the M0 model. The next three simulated data sets, called B, C, and D, were obtained by using the *R* rate matrix of simulation A and adding randomly drawing double and triple rates from normal distributions with varying mean and variances (simulation B: N(0.0001,0.03); simulation C: N(0.001,0.1); simulation D: N(0.1,0.1)). This lead to approximately 11%, 30%, and 39% of double and triple substitutions for these three simulation schemes, respectively. Moreover, we complemented our data sets by using an empirical rate matrix derived by Kosiol et al. (2007), which is based on empirical data from the Pandit database. This data set that we refer to as ECM is composed of approximately 25% of double and triple substitutions.

The expected values of the *ω* parameter for the data sets A, B, C, and D were initially set to 1. This was not possible for the ECM schemes, which has an unknown *ω* value estimated by Kosiol et al. (2007) to be close to 0.3. We nevertheless created four other rate matrices by multiplying every substitution rate leading to a nonsynonymous change by 0.1, 0.5, 2, and 10. Given these rate matrices *R* (one for each factor), we randomly created 50 random trees with the R package ape (function rtree with parameters br = rlnorm, mean = 0.41, SD = 0.34; Paradis et al. 2004), each composed of 15 sequences and simulated an alignment composed of 150 codons. The mean and variance of the total branch length estimated under the M0 model for the five simulated data sets describe the sequence divergence considered by our analysis (see supplementary table S2, Supplementary Material online). We therefore simulated 5 simulation schemes, each with 5 different *ω* values and replicated this 50 times for a total of 1,250 simulated data sets. For data set A, we used the original evolver software (Yang 2007), whereas we implemented a modified version of evolver package that takes as an input a 64 × 64 *R* matrix. We checked the effects of the small alignment length by repeating the simulations with 3,500 codons for the simulations A, B, and ECM for *ω* values 0.5 and 2.0.

Beside the simulated data sets, we assessed our new model on known protein-coding genes that included the following: 1) *β-Globin* sequences containing 144 codons for 17 vertebrate species extracted from GenBank; 2) *rbcL* sequences from plants containing 447 codons for 20 species; and 3) *pepC* sequences also from plants containing 437 codons for 20 species. The two plant data sets were part of larger studies (Christin et al. 2007, 2008) and included several hundreds of taxa. We randomly selected 20 species from the published aligned matrices of these genes to save computational time. We additionally evaluated the effects of the different KCM models on 100 empirical data sets (supplementary table S3, Supplementary Material online) randomly selected from Selectome database (Moretti et al. 2013) and compared the fit of these models with M0 and MEC. These data sets are representative of typical data sets analyzed by codon models because the *ω* values ranged from 0.0054 to 8.66745, whereas the number of sequences and length of alignment is typical of current data sets (supplementary table S1, Supplementary Material online).

The biological relevance of the *Q* matrices returned by the best KCM variant was assessed using the almost invariant sites approach (Kosiol et al. 2004) as implemented in the AIS software. This approach attempts to group codons into classes that have high probabilities of change within each class while having small probability of change between different classes. The analyses were performed with either 20 or 7 classes of codons representing the 20 amino acids or 7 physicochemical properties of amino acids.

All estimations of maximum likelihood under the KCM model were done in a modified version of the CodeML software that is available at the address www.unil.ch/phylo/Bioinformatics (last accessed July 11, 2014).

## Supplementary Material

Supplementary figure S1–S8 and tables S1–S4 are available at *Molecular Biology and Evolution* online (http://www.mbe.oxfordjournals.org/).

Supplementary Data
