# Supplementary material for: A Generalized Mechanistic Codon Model
Source: Mol Biol Evol. 2014 Jun 23;31(9):2528–41. doi: 10.1093/molbev/msu196 (PMC4137716; doi:10.1093/molbev/msu196)
Supplement: Supplementary Data [file supp_31_9_2528__index.html]

A Generalized Mechanistic Codon Model — A Generalized Mechanistic Codon Model — Supplementary Data 

# A Generalized Mechanistic Codon Model

## Supplementary Data

file

**Files in this Data Supplement:**

- Supplementary Data - pdf file
