# Supplementary material for: A Generalized Mechanistic Codon Model
Source: Mol Biol Evol. 2014 Jun 23;31(9):2528–41. doi: 10.1093/molbev/msu196 (PMC4137716; doi:10.1093/molbev/msu196)
Supplement: Supplementary Data [file supp_msu196_suppMat_Zaherietal.pdf]

# Supplementary Material online:
# A Generalized Mechanistic Model for double and triple codon substitution

Maryam Zaheri[1,2,*], Linda Dib[1,2,*] and Nicolas Salamin[1,2]

[1]Department of Ecology and Evolution, University of Lausanne, 1015 Lausanne, Switzerland

[2]Swiss Institute of Bioinformatics, Quartier Sorge, 1015 Lausanne, Switzerland

[*]co-first authors

Corresponding author: Nicolas Salamin, Department of Ecology and Evolution, Biophore, University of Lausanne, 1015 Lausanne, Switzerland; tel: +41 21 692 4154; fax: +41 21 692 4265; email: nicolas.salamin@unil.ch

Keywords: codon models, phylogenetics, multiple substitutions, positive selection, Markov model, Kronecker product

Running head: Generalized codon model

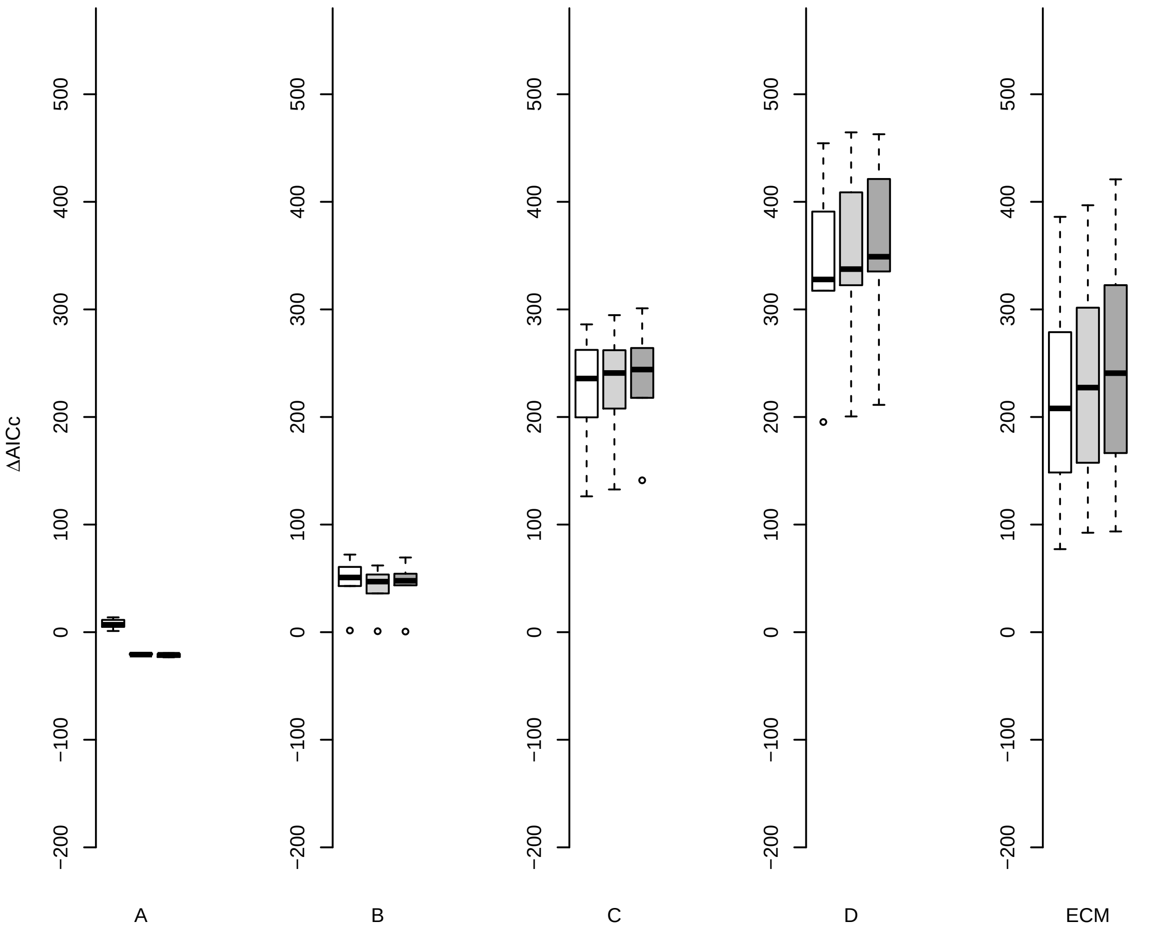


Supplementary fig. 1: Delta AICc plots comparing the performance of the $KCM_{19x}$ model to *M0* model on 5 simulated data sets. Here, we compared the models using three frequency modes: $F1/61$ (white), $F3 \times 4$ (light grey) and $F61$ (dark grey) as mentionned in Yang and Bielawski (2000).

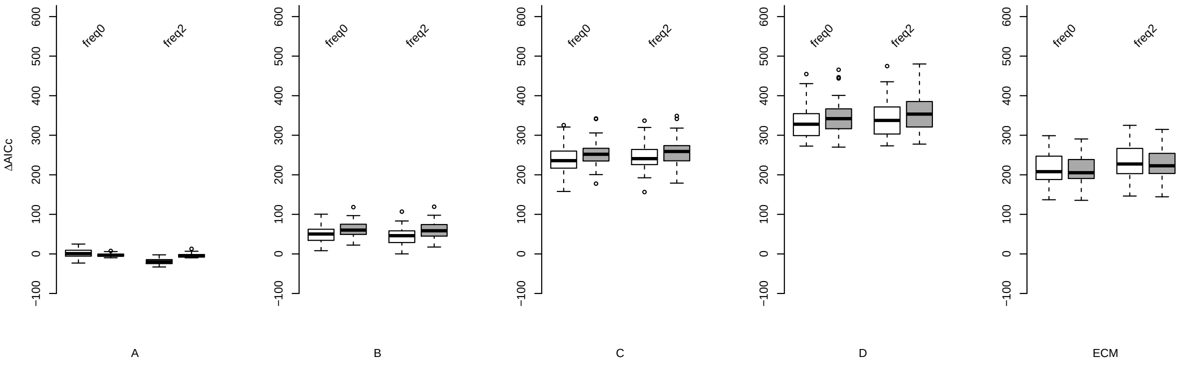


Supplementary fig. 2: Delta AICc plots comparing the performance of the $KCM_{7x}$ and $KCM_{19x}$ models to *M0* model on 5 simulated data sets for ω factor = 1. We compared the models using two frequency modes: the first one where all frequencies are equal ($F1/61$) and the second where we used the products of the observed nucleotide frequencies at each of the 3 codon positions ($F3 \times 4$; Yang and Bielawski, 2000).

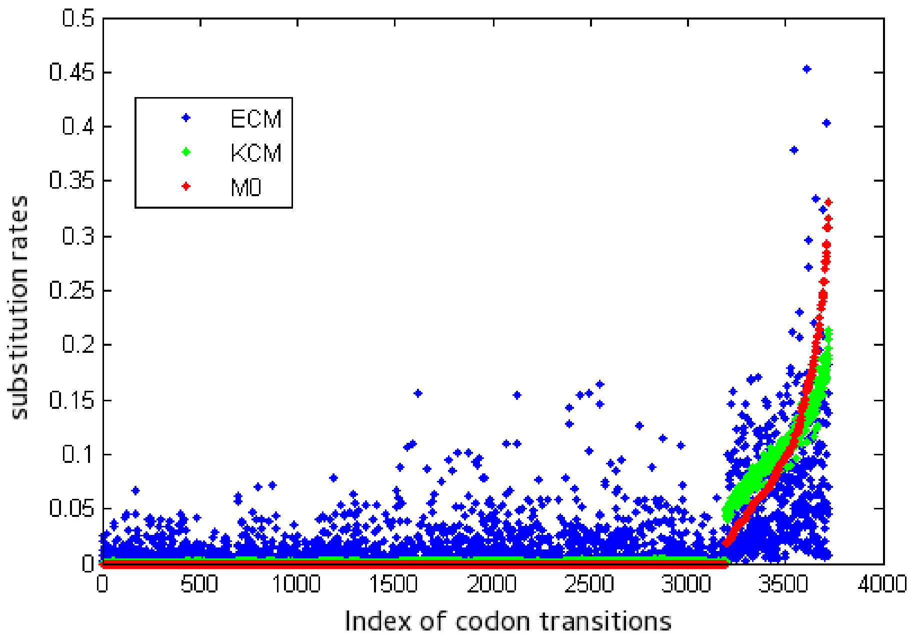


Supplementary fig. 3: Plot of substitution rates per codon of $KCM_{19x}$, *ECM* and *M0* models for data simulated under the *M0* model. The x-axis is the index of the 3,660 rates of transition between codons obtained from the rate matrices estimated by the different models, while the y-axis is the rate of substitution per codon. The substitution rates from the *M0* model is used as the reference distribution. The median of the 50 simulations for each entity of the $61 \times 61$ rate matrix are plotted for *M0* (red), $KCM_{19x}$ (green) and *ECM* (blue) models. The simulations are realized under the *M0* model with $\omega = 1$. The points are sorted according to the entities of the *M0* substitution rate matrix. The Euclidean distance between $KCM_{19x}$ substitution rates and the *M0* ones was 1.005, while for *ECM*the Euclidian distance with *M0* rates was 2.281.

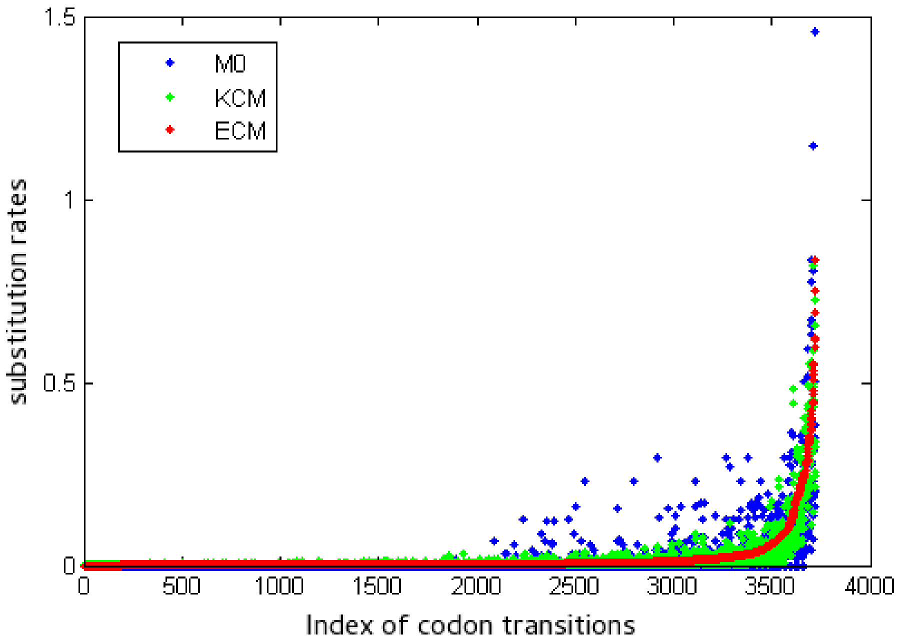


Supplementary fig. 4: Plot of substitution rates per codon of $KCM_{19x}$ *ECM* and *M0* models for data simulated under the *ECM* model. The x-axis is the index of the 3,660 rates of transition between codons obtained from the rate matrices estimated by the different models, while the y-axis is the rate of substitution per codon. The substitution rates from the *ECM* model is used as the reference distribution. The median of the 50 simulations for each entity of the $61 \times 61$ rate matrix are plotted for *ECM* (red), $KCM_{19x}$ (green) and *M0* (blue). The simulations are realized under the *ECM* model with $\omega$ factor $= 1$. The points are sorted according to the entities of the *ECM* substitution rate matrix. The Euclidean distance between $KCM_{19x}$ substitution rates and the *ECM* ones was 1.812, while the Euclidean distance between *M0* substitution rates and the *ECM* ones was 2.789.

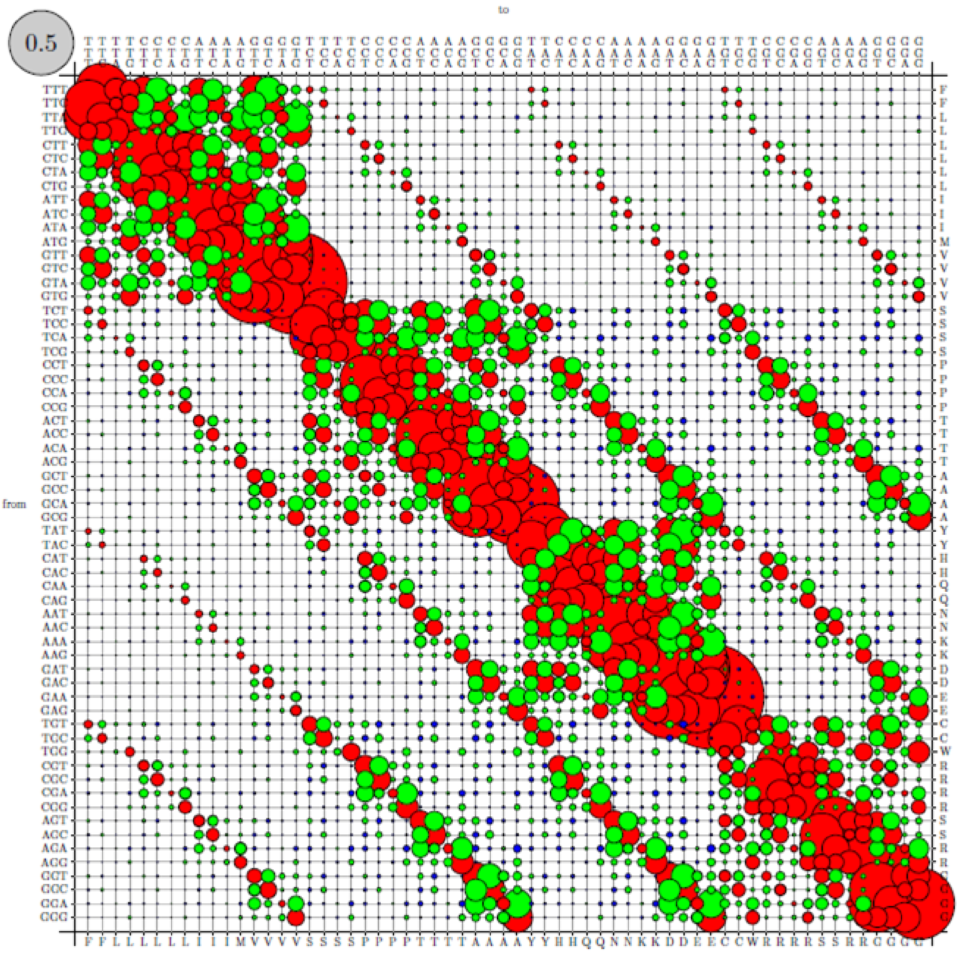


Supplementary fig. 5: Bubble plot of the $KCM_{19x}$ model for the β-*globin* data sets. Single, double and triple nucleotide substitutions are in red, green and blue respectively. Codons are according to Urbina et al (2006).

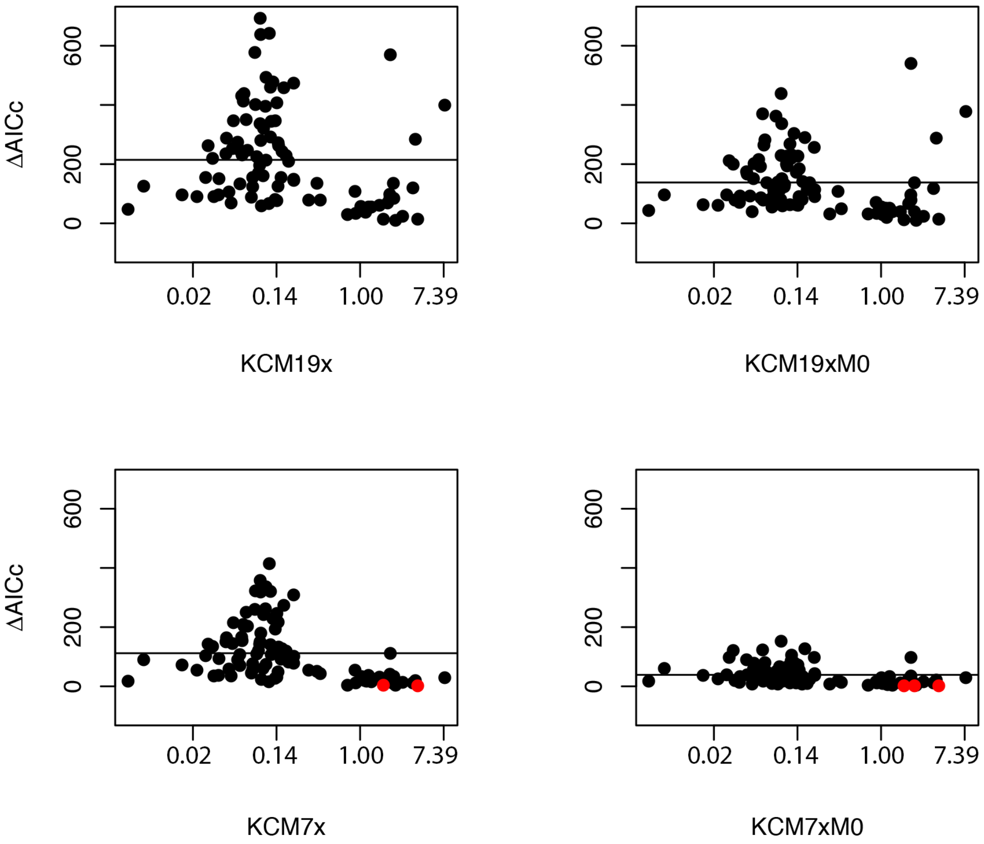


Supplementary fig. 6: Delta AICc plots comparing the performance of the *M0* model to all variants of the *KCM* model on 100 empirical data sets randomly selected from the Selectome data base. For each plot, a black horizontal line is draw for the mean delta AICc value of the empirical dataset. Note that for this analysis we considered a frequency vector calculated using the products of the observed nucleotide frequencies at each of the 3 codon positions ($F3 \times 4$ Yang and Bielawski, 2000). Empirical data sets with delta AICc $< 4$ are shown in red.

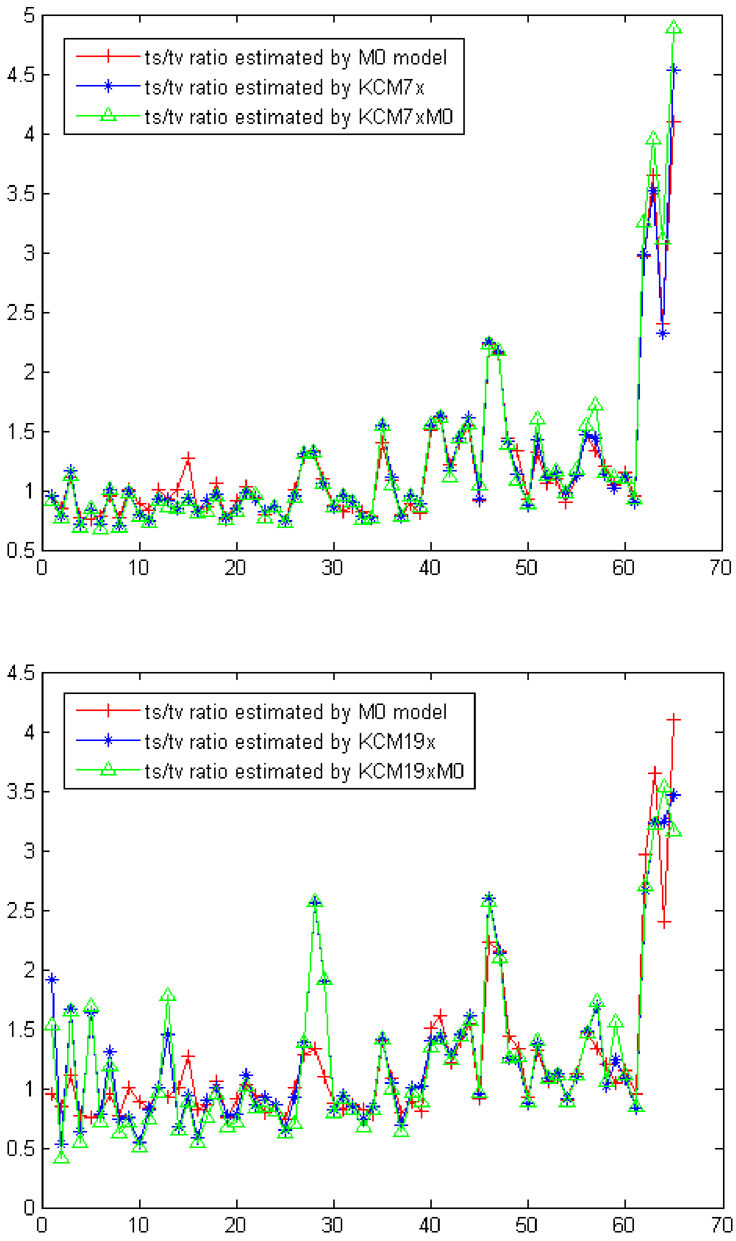


Supplementary fig. 7: Estimation of the average rate of transition and transversion for the empirical data sets selected from the Selectome database. We only used the 65 data sets that did not contain gaps to create this plot. The rates are estimated from the nucleotide substitution matrix (the rates for the three matrices of $KCM_{19x}$ are averaged) based on the formula described in Yang (2006).

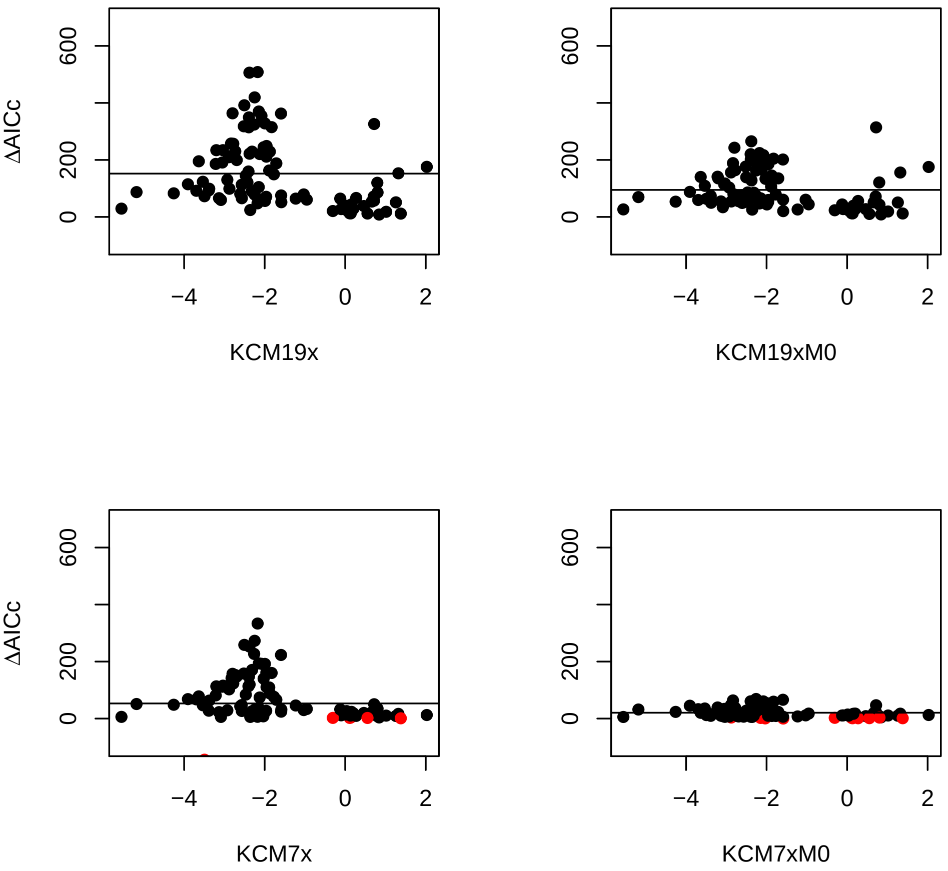


Supplementary fig. 8: Delta AICc plots comparing the performance of the *M0* model to all variants of the *KCM* model on 100 empirical data sets randomly selected from the Selectome data base. For each plot, a black horizontal line is draw for the mean delta AICc value of the empirical dataset. Note that for this analysis we considered a frequency vector with equal codon frequencies ($F1/61$ Yang and Bielawski, 2000). Empirical data sets with delta AICc $<4$ are shown in red.

# Supplementary tables

Supplementary table 1: Comparison of the estimation of the ω factors for data simulated with 150 and 3500 codons. The median and mean ω estimated for different alignment lengths is reported for simulations A, B and ECM with factors on the ω parameter of 0.5 and 2.0.

| ω factor | Simulations | Mean | | Variance | |
|---|---|---|---|---|---|
| | | 150 codons | 3500 codons | 150 codons | 3500 codons |
| 0.5 | A | 0.519 | 0.514 | 1.606e-2 | 1.063e-3 |
| | B | 0.395 | 0.369 | 7.514e-3 | 3.926e-4 |
| | ECM | 0.042 | 0.035 | 1.149e-4 | 1.954e-6 |
| 2.0 | A | 2.614 | 2.206 | 3.297e-1 | 1.504e-2 |
| | B | 1.580 | 1.500 | 4.351e-1 | 1.792e-2 |
| | ECM | 0.135 | 0.130 | 8.091e-4 | 4.271e-5 |

Supplementary table 2: Mean, variance, minimum and maximum total branch lengths for a whole alignment (450 codons) for the simulated data sets based on the settings of simulation A with ω factor set to 0.1, 0.5, 1, 2 and 10. The large variance obtained for ω factor = 0.1 is due to a single simulation leading to large divergence between sequences (i.e. the maximum value of 110.808).

| ω factor | Total branch lengths | | | |
|---|---|---|---|---|
| | Mean | Variance | Minimum | Maximum |
| 0.1 | 54.806 | 176.515 | 36.651 | 110.808 |
| 0.5 | 41.582 | 13.836 | 36.397 | 54.520 |
| 1.0 | 38.285 | 8.503 | 32.229 | 47.488 |
| 2.0 | 36.981 | 5.490 | 31.759 | 42.425 |
| 10.0 | 38.365 | 6.484 | 32.723 | 42.823 |

Supplementary table 3: List of the 100 empirical data sets selected from the Selectome database. The value of ω was estimated with the *M0* model.

| Family | ω | Number of species | Alignment length |
|---|---|---|---|
| ENSGT00390000007638.Euteleostomi.001 | 8.66745 | 10 | 229 |
| ENSGT00530000064869.Euteleostomi.001 | 6.462 | 8 | 98 |
| ENSGT00390000016329.Euteleostomi.001 | 3.923 | 12 | 88 |
| ENSGT00400000024605.Euteleostomi.001 | 2.744 | 9 | 163 |
| ENSGT00390000016066.Euteleostomi.001 | 2.656 | 14 | 178 |
| ENSGT00410000029170.Euteleostomi.001 | 2.494 | 9 | 62 |

Continued on next page

**Supplementary table 4 – continued from previous page**

| Family | ω | Number of species | Alignment length |
|---|---|---|---|
| ENSGT00390000018599.Euteleostomi.001 | 2.408 | 8 | 168 |
| ENSGT00680000100237.Euteleostomi.001 | 2.397 | 13 | 404 |
| ENSGT00530000064897.Euteleostomi.001 | 2.332 | 9 | 144 |
| ENSGT00410000026913.Euteleostomi.001 | 2.313 | 13 | 384 |
| ENSGT00680000100326.Euteleostomi.001 | 2.233 | 9 | 105 |
| ENSGT00410000028440.Euteleostomi.001 | 2.217 | 8 | 69 |
| ENSGT00530000065014.Euteleostomi.001 | 2.198 | 7 | 143 |
| ENSGT00530000064466.Euteleostomi.001 | 2.113 | 11 | 64 |
| ENSGT00540000072033.Euteleostomi.001 | 2.014 | 23 | 636 |
| ENSGT00680000100122.Euteleostomi.001 | 1.933 | 25 | 428 |
| ENSGT00680000101349.Euteleostomi.001 | 1.857 | 16 | 166 |
| ENSGT00540000072033.Primates.001 | 1.794 | 24 | 567 |
| ENSGT00530000062931.Primates.005 | 1.775 | 28 | 72 |
| ENSGT00630000089916.Euteleostomi.001 | 1.612 | 48 | 64 |
| ENSGT00680000100179.Euteleostomi.001 | 1.319 | 22 | 401 |
| ENSGT00400000022365.Primates.001 | 1.226 | 16 | 133 |
| ENSGT00390000003272.Euteleostomi.001 | 1.214 | 17 | 287 |
| ENSGT00390000003272.Primates.001 | 1.211 | 17 | 273 |
| ENSGT00650000093893.Euteleostomi.001 | 1.187 | 19 | 139 |
| ENSGT00680000100090.Euteleostomi.001 | 1.171 | 41 | 579 |
| ENSGT00390000012484.Primates.001 | 1.108 | 33 | 229 |
| ENSGT00700000104581.Primates.001 | 1.027 | 20 | 491 |
| ENSGT00610000086906.Euteleostomi.001 | 1.026 | 17 | 190 |
| ENSGT00400000022365.Euteleostomi.001 | 1.007 | 16 | 128 |
| ENSGT00530000064497.Primates.001 | 0.878 | 20 | 142 |
| ENSGT00390000012671.Euteleostomi.001 | 0.451 | 26 | 310 |
| ENSGT00700000104246.Primates.002 | 0.447 | 27 | 90 |
| ENSGT00390000001323.Euteleostomi.001 | 0.384 | 23 | 225 |
| ENSGT00390000006080.Euteleostomi.001 | 0.313 | 32 | 847 |
| ENSGT00390000014593.Euteleostomi.001 | 0.302 | 25 | 232 |
| ENSGT00650000093105.Euteleostomi.007 | 0.286 | 27 | 516 |
| ENSGT00390000000252.Euteleostomi.001 | 0.237 | 25 | 268 |
| ENSGT00390000003056.Euteleostomi.001 | 0.234 | 18 | 417 |
| ENSGT00510000047079.Euteleostomi.001 | 0.228 | 41 | 380 |
| ENSGT00440000039958.Euteleostomi.001 | 0.222 | 19 | 518 |
| ENSGT00570000078866.Euteleostomi.003 | 0.206 | 30 | 488 |
| ENSGT00550000074403.Euteleostomi.003 | 0.202 | 45 | 406 |
| ENSGT00390000017791.Euteleostomi.001 | 0.190 | 42 | 204 |
| ENSGT00390000011439.Euteleostomi.001 | 0.190 | 37 | 343 |
| ENSGT00390000002149.Euteleostomi.003 | 0.184 | 38 | 370 |
| ENSGT00390000018632.Euteleostomi.001 | 0.175 | 19 | 912 |
| ENSGT00390000001055.Euteleostomi.001 | 0.170 | 47 | 217 |
| ENSGT00550000075001.Euteleostomi.001 | 0.170 | 40 | 450 |
| ENSGT00530000063224.Euteleostomi.003 | 0.165 | 33 | 595 |

Continued on next page

**Supplementary table 4 – continued from previous page**

| Family | ω | Number of species | Alignment length |
|---|---|---|---|
| ENSGT00530000062962.Euteleostomi.002 | 0.162 | 47 | 366 |
| ENSGT00390000000377.Euteleostomi.001 | 0.156 | 33 | 824 |
| ENSGT00390000016028.Euteleostomi.006 | 0.147 | 39 | 401 |
| ENSGT00390000011804.Euteleostomi.001 | 0.142 | 30 | 159 |
| ENSGT00680000099596.Euteleostomi.004 | 0.139 | 32 | 635 |
| ENSGT00390000018208.Euteleostomi.001 | 0.136 | 26 | 168 |
| ENSGT00390000010568.Euteleostomi.001 | 0.135 | 43 | 296 |
| ENSGT00550000074304.Euteleostomi.004 | 0.135 | 36 | 541 |
| ENSGT00550000074424.Euteleostomi.001 | 0.132 | 46 | 503 |
| ENSGT00390000004323.Euteleostomi.001 | 0.131 | 33 | 268 |
| ENSGT00390000002563.Euteleostomi.002 | 0.129 | 42 | 593 |
| ENSGT00510000048936.Euteleostomi.001 | 0.129 | 23 | 429 |
| ENSGT00390000008178.Euteleostomi.001 | 0.128 | 29 | 722 |
| ENSGT00630000089834.Euteleostomi.001 | 0.127 | 36 | 936 |
| ENSGT00660000095648.Euteleostomi.001 | 0.126 | 18 | 419 |
| ENSGT00390000004313.Euteleostomi.001 | 0.121 | 28 | 185 |
| ENSGT00680000099772.Euteleostomi.001 | 0.120 | 29 | 389 |
| ENSGT00510000047481.Euteleostomi.001 | 0.114 | 36 | 583 |
| ENSGT00530000063670.Euteleostomi.002 | 0.113 | 29 | 550 |
| ENSGT00510000047499.Euteleostomi.001 | 0.112 | 33 | 461 |
| ENSGT00660000095322.Euteleostomi.002 | 0.108 | 40 | 949 |
| ENSGT00390000001969.Euteleostomi.003 | 0.106 | 29 | 804 |
| ENSGT00600000084107.Euteleostomi.001 | 0.099 | 41 | 853 |
| ENSGT00530000063122.Euteleostomi.003 | 0.091 | 34 | 572 |
| ENSGT00530000063319.Euteleostomi.001 | 0.077 | 16 | 580 |
| ENSGT00530000063473.Euteleostomi.001 | 0.074 | 30 | 608 |
| ENSGT00390000014093.Euteleostomi.002 | 0.073 | 36 | 443 |
| ENSGT00620000087732.Euteleostomi.003 | 0.066 | 37 | 499 |
| ENSGT00550000074477.Euteleostomi.002 | 0.066 | 44 | 522 |
| ENSGT00390000014517.Euteleostomi.001 | 0.066 | 33 | 232 |
| ENSGT00390000016435.Euteleostomi.001 | 0.065 | 36 | 527 |
| ENSGT00390000005712.Euteleostomi.001 | 0.064 | 37 | 638 |
| ENSGT00550000074298.Euteleostomi.001 | 0.063 | 35 | 669 |
| ENSGT00530000063581.Euteleostomi.002 | 0.062 | 34 | 422 |
| ENSGT00390000002779.Euteleostomi.001 | 0.058 | 38 | 516 |
| ENSGT00670000097509.Euteleostomi.001 | 0.056 | 33 | 488 |
| ENSGT00390000016168.Euteleostomi.001 | 0.052 | 48 | 688 |
| ENSGT00390000020883.Euteleostomi.001 | 0.051 | 18 | 669 |
| ENSGT00510000047893.Euteleostomi.001 | 0.051 | 29 | 420 |
| ENSGT00660000095228.Euteleostomi.002 | 0.047 | 31 | 412 |
| ENSGT00390000004653.Euteleostomi.003 | 0.046 | 35 | 277 |
| ENSGT00530000062914.Euteleostomi.003 | 0.039 | 40 | 386 |
| ENSGT00560000076730.Euteleostomi.006 | 0.038 | 26 | 462 |
| ENSGT00560000076885.Euteleostomi.003 | 0.035 | 48 | 302 |

Continued on next page

**Supplementary table 4 – continued from previous page**

| Family | ω | Number of species | Alignment length |
|---|---|---|---|
| ENSGT00660000095340.Euteleostomi.004 | 0.029 | 45 | 998 |
| ENSGT00650000093277.Euteleostomi.004 | 0.027 | 38 | 311 |
| ENSGT00550000074326.Euteleostomi.004 | 0.022 | 41 | 318 |
| ENSGT00390000004852.Euteleostomi.001 | 0.014 | 49 | 183 |
| ENSGT00660000095549.Euteleostomi.001 | 0.013 | 31 | 428 |
| ENSGT00550000075088.Euteleostomi.001 | 0.005 | 41 | 158 |
